# Exploring and exploiting the connection between mitochondria and the virulence of human pathogenic fungi

**DOI:** 10.1080/21505594.2017.1414133

**Published:** 2018-03-01

**Authors:** Surbhi Verma, Viplendra P. S. Shakya, Alexander Idnurm

**Affiliations:** a Department of Biochemistry, University of Utah School of Medicine, Salt Lake City, Utah, USA; b School of BioSciences, University of Melbourne, Parkville, VIC, Australia

**Keywords:** *Aspergillus fumigatus*, azole antifungal, *Candida*, *Cryptococcus*, heme, iron, mitochondria, pathogenicity, recombination

## Abstract

Mitochondria are best known for their role in the production of ATP; however, recent research implicates other mitochondrial functions in the virulence of human pathogenic fungi. Inhibitors of mitochondrial succinate dehydrogenase or the electron transport chain are successfully used to combat plant pathogenic fungi, but similar inhibition of mitochondrial functions has not been pursued for applications in medical mycology. Advances in understanding mitochondrial function relevant to human pathogenic fungi are in four major directions: 1) the role of mitochondrial morphology in virulence, 2) mitochondrial genetics, with a focus on mitochondrial DNA recombination and mitochondrial inheritance 3) the role of mitochondria in drug resistance, and 4) the interaction of mitochondria with other organelles. Collectively, despite the similarities in mitochondrial functions between fungi and animals, this organelle is currently an under-explored potential target to treat medical mycoses. Future research could define and then exploit those mitochondrial components best suited as drug targets.

## Introduction

Mitochondria are double membrane-bound organelles that serve as the powerhouses of cells, with one of their primary roles being to generate the cell's energy currency, adenosine triphosphate (ATP). However, mitochondria also perform other diverse functions in the cell: they are the hub for respiratory and other metabolic activities, being the home for major aerobic metabolic cycles such as the citric acid cycle (TCA) and the electron transport chain (ETC), the subcellular location of the synthesis of certain metabolites such as amino acids [1] and for the biosynthesis of heme and the regulation of cellular iron homeostasis [2], and provide feedback on the cellular status to the nucleus. The earliest reports about mitochondria trace back to the 19^th^ century, with an initial description in 1890 by Altman as “bioblasts” described as “elementary organisms” living inside cells and carrying important functions [3]. Views on the roles and importance of mitochondria have expanded since the early 1900's from the discoveries of ATP by Lohmann in 1929 and the TCA cycle by Krebs and Johnson in 1937, the later formulation of the chemiosmotic coupling hypothesis by Mitchell in 1961, to the implication of mitochondrial function in human pathology [4], the association of mitochondria to drug resistance in yeast [5], mitochondrial DNA mutation as a cause of human diseases [6–7], the input of mitochondria in apoptosis [8], mitochondria involvement in aging related disorders such as Alzheimer's and Parkinson's diseases [9–10], and mitochondrial inputs in metabolic disorders such as diabetes [11]. Despite over a century of research on these organelles, new discoveries are still being made. One recent example was defining a new function in aspartate synthesis to maintain cell proliferation that expanded the role of the ETC, which until then had been limited to ATP synthesis [12].

Among the multiple functions of mitochondria, one key role is in influencing host-pathogen interactions, from both the host's and the pathogen's perspectives [13–16]. The importance of mitochondrial function beyond respiration is illustrated by mitochondrial functions in some parasitic eukaryotic organisms. For instance, despite no respiration, and at some points during history considered to have no mitochondria, species in genera like *Entamoeba, Giardia* and *Trichomonas* have remnants or modified forms of mitochondria [17], highlighting the essential requirement of these organelles for the compartmentalization of biochemical reactions within cells. The microsporidia, an early branch in fungal evolution and obligate pathogens, also have highly reduced mitochondria, which are believed to be essential for the synthesis of iron-sulfur clusters [18]. Thus, even with extreme levels of genome reduction and long evolutionary time, mitochondria are still maintained in these groups of pathogens. Human fungal pathogens that are the causes of life-threatening diseases such as those in the *Aspergillus, Candida* and *Cryptococcus* genera are aerobic in nature, and thus there is an essential role of mitochondrial morphology, genetics or metabolism for their survival in the environment as well as during host infection where oxygen concentrations can vary within different host tissues [19–22]. Fungal pathogens of both humans and plants require a functional mitochondrial genome and nuclear-encoded mitochondrially-localized proteins during the establishment of infection or in virulence [23–26]. Here, we will address the pathogen's side of the interaction, with our major focus on the role of virulence traits associated with mitochondria in human pathogenic fungi.

Over the course of evolution mitochondria have moved most of their genome contents into the nucleus, thus most mitochondrial proteins are encoded in the nucleus, translated by cytosolic ribosomes, and subsequently imported into the mitochondria. Along with tRNAs, ribosomal RNA and ribosome-associated proteins are required for mitochondrial translation. The proteins encoded in mitochondrial genomes are mostly the core components of oxidative phosphorylation, with seven proteins encoded in the mitochondrial genome of the model yeast *Saccharomyces cerevisiae* or the related human pathogen *Candida glabrata* [27–29]. The mitochondrial genomes of the pathogens *Cryptococcus deneoforman*s and *C. deuterogattii* encode thirteen proteins (reviewed in [30]). The mitochondrial genomes of species in *Aspergillus* and *Penicillium* encode fourteen proteins, where in most cases gene synteny is conserved [31].

Fungi are eukaryotes and when compared to bacterial pathogens they are difficult to target with pharmaceutical agents due to their genomic complexity and biochemical similarities to humans [32]. There are at least two major reasons to focus on mitochondria in pathogenic fungi. 1) Drug resistance is a major problem in treatment of many fungal infections and some drug resistance mechanisms have a mitochondrial input. For instance, the target enzyme for the most commonly used antifungals, the azoles, requires a heme cofactor that is synthesized in the mitochondria. 2) There is clear evidence from fungal pathogens that mitochondria are part of their armory to cause disease. Mitochondrial components are conceivably good substrates as targets of new drugs and for understanding the spread and emergence of virulence of fungal pathogens (reviewed in references [33–34], and in this article). Exploring mitochondrial drug targets for combating fungal infections is a promising and a feasible avenue, as is evident by auranofin, a rheumatoid arthritis drug that was recently “repurposed” as an antifungal drug [35]. The mode of action for auranofin is through targeting Mia 40 (mitochondrial intermembrane space import and assembly protein 40), an essential mitochondrial protein required for the oxidation of cysteine-containing proteins in the mitochondria. Auranofin inhibits import of Cmc1 (cytochrome c oxidase biogenesis factor), a mitochondrial protein substrate imported by the Mia40/Erv1 pathway. Auranofin is effective against *C. neoformans* in a *Caenorhabditis elegans* infection model and is a candidate for treating fungal infections in humans. Hence, exploring mitochondrial antifungal targets is an important and promising research direction yet with little attention directed to it at present.

## Mitochondrial dynamics and morphology influence fungal pathogenicity

Despite how textbooks depict them, as seemingly static structures, mitochondria are highly dynamic organelles as they undergo fission and fusion to maintain function during both normal and stress conditions [36–37]. Fission is required for the production of new mitochondria, autophagy of old or damaged mitochondria, and for the process of apoptosis. The fusion of mitochondria, on the other hand, helps the cell cope with increased energy demands and stress conditions, by forming a network of mitochondria that can share resources and their genetic material amongst each other for survival. In *Saccharomyces cerevisiae* the fission and fusion of mitochondria are well characterized, mediated through Dnm1 and Fzo1, respectively, which both belong to the GTPase family of proteins and are conserved and characterized in other fungi, fruit flies and mammals [36–38].

*Cryptococcus* species cause fatal lung infections and meningitis in humans and animals, and they are obligate aerobes with a strict requirement for actively respiring mitochondria such that no “petite” forms are observed during in vitro culturing (petite strains are respiration deficient, as they lose major portions or all of their mitochondrial genomes, so produce small colonies in species like *S. cerevisiae*). In this review we follow the new *Cryptococcus* species nomenclature [39]. For clarification, *C. neoformans* var. *grubii* and *C. neoformans* var. *neoformans* have been formally described as separate species *C. neoformans* and *C. deneoformans*, respectively. For *C. gattii* five species are described: *C. gattii* (VGI; nomenclature based on *C. neoformans* var. *gattii* lineage I), *C. deuterogattii* (VGII), *C. bacillisporus* (VGIII), and *C. tetragattii* (VGIV) and *C. decagattii*. Species in the “*C. gattii* complex” are characterized by infecting immune competent individuals.

Properties of strains of *C. deuterogattii* have been linked to mitochondrial morphology and dynamics [19,40]. Specifically, the most virulent strains responsible for an outbreak of cryptococcosis, starting on Vancouver Island in the late 1990s, feature tubular mitochondria that are correlated with an enhanced intracellular parasitism rate in a macrophage cell line. Moreover, gene expression studies show that up-regulation of *FZO1* correlates with the tubular mitochondrial morphology observed under stress conditions, with one of these stresses being exposure to reactive oxygen species like H_2_O_2_ [41].

In *Candida albicans*, loss of *FZO1* has pleiotropic effects on the cell [42]. Mutants for *FZO1* have mitochondrial fusion defects along with a significant loss in mitochondrial genome maintenance. Consequences associated with a suite of functional and metabolic anomalies include perturbation of iron assimilation and metabolism, increased susceptibility to peroxide stress due to impairment in the Hog1 signaling pathway and increased susceptibility to azole drugs, mainly due to missorting of Cdr1 efflux pumps to the vacuole. In another fungal pathogen, *Aspergillus fumigatus*, mitochondrial dynamics and morphology affect drug resistance and virulence potential [38]. Interestingly, the fission mutants for this fungus, *fis1*Δ, *mdv1*Δ and *dnm1*Δ, have diminished growth on complex media and aberrant mitochondrial morphology compared to wild type cells. Surprisingly all three mutants showed increased resistance to azole drugs like posaconazole and voriconazole, which is attributed to altered lanosterol 14α-demethylation (Erg11) activity, an enzyme involved in ergosterol biosynthesis. When examined for virulence, these azole-resistant and slow growing fission mutants have normal or slightly decreased virulence in a *Galleria mellonella* (wax moth) infection model. However, a strain of *A. fumigatus* with a conditional mutation in the essential gene *Mgm1*, which encodes another GTPase required for mitochondrial fusion, was avirulent in the same *G. mellonella* infection model [38].

It is also worth noting that genes associated with mitochondrial morphology and maintenance also play a crucial role in governing growth and virulence in plant pathogens, and several examples are provided in the following sections. In rice blast fungus *Pyricularia* (*Magnaporthe*) *oryzae*, which is a major agricultural pathogen associated with most of the rice crop losses around the world, mutation of the gene encoding fission associated outer mitochondrial membrane protein MoFis1 affects conidiation, colony growth and virulence of the pathogen [43]. Other genes associated with mitochondria in *P. oryzae* affect growth, conidiation, pathogenicity and the formation of appressoria, specialized infectious cell structures with flattened hyphae giving rise to infection pegs that can penetrate through the host cuticle into epidermal cells using turgor pressure [44]. Loss of the mitochondrial fatty acid metabolism associated gene enoyl-CoA hydratase (*Ech1*) affects growth on carbon sources and impacts mitochondrial morphology, cell integrity and pathogenicity. Mutant strains of the *Ech1* gene have more punctate mitochondria compared to the fused tubular mitochondrial morphology observed in wild type cells. The pathogenicity of the *ech1*Δ strains was compromised, as they could not cause lesions on barley and rice leaf blades. The *ech1*Δ mutant has reduced melanization, with about twenty percent of the appressoria able to penetrate for the leaf, and even then they remain localized to the primary site of infection, whereas wild type appressoria showed full penetration and secondary host tissue invasion. Another example is from the maize pathogen *Ustilago maydis*, in which loss of the fission-associated gene *DNM1* can impact the mitochondria and pathogenicity of the fungus [25]. Plants infected with *U. maydis dnm1*Δ strains showed reduced disease symptoms compared to those infected by the wild type.

Given that fungi diverged from plants well before they did from animals, the potential specificity to target mitochondrial functions in agriculturally-relevant fungal pathogens vs. their plant hosts is of high relevance. Work on mitochondrial functions from plant pathogens highlights the potential to target components of this organelle for disease control. Indeed, succinate dehydrogenase inhibitor (SDHI) fungicides that target the mitochondrial enzyme succinate dehydrogenase have been used in agriculture since the 1960s. These fungicides specifically block the mitochondrial complex II ubiquinone-binding sites and hence inhibit fungal respiration. Carboxin is one of the early SDHI, which has been used since the 1960s, and is active against plant pathogenic basidiomycetes such as *U. maydis* [45]. Since then, new generations of SDHIs were developed and are used more broadly to protect crops [46]. Another group of fungicides targeting the mitochondrial electron transport chain are the strobilurins, belonging to a group of respiratory chain complex III inhibitors [47]. However there is an increasing problem of acquired resistance evolving against these fungicides which warrants the development of new active fungicidal compounds that target the pathogens' respiratory hub [48].

Although such inhibitors of mitochondria function have been developed and effectively used against plant pathogens, such a major leap is still awaited in medically-important fungi where mitochondrial functions are equally vulnerable to chemical inhibition but more difficult to be safely targeted considering the closer evolutionary relationship of fungi to their human hosts. However, the discovery of the chemical ME1111, which inhibits *Trichophyton* species which are agents of nail disease, as a succinate dehydrogenase inhibitor, without inhibiting human mitochondria, suggests that such targeting may be a valid direction [49]. Another exciting example is the discovery of the chemical F901318, which inhibits fungal dihydroorotate dehydrogenase with greater specificity over the human homolog, and is active against a subset of ascomycete human pathogens including *A. fumigatus* in mouse models [50]. In some cases even the inhibitors for conserved mitochondrial proteins can be a potential therapeutic, provided they have higher affinity for fungal counterparts compared to mammalian proteins. For example, the chemical Inz-1 inhibits complex III of the mitochondrial respiratory chain with higher selectivity for *C. albicans* than the human complex [51]. However, as there are few explored targets a broader understanding of mitochondrial function in pathogens will help in overcoming this barrier.

There are fungal-specific proteins with both mitochondrial functions and roles in virulence, such as Goa1, Mcu1 and components of ERMES with no homologs present in mammals, which makes them attractive drug targets [33]. Mcu1 (multiple carbon source utilizer 1) is a mitochondrial protein required by *C. albicans* for virulence in a murine model of disease [52]. Mcu1 is required by *C. albicans* for utilization of *N*-acetylglucosamine, a cell wall component and an important constituent in the mammalian gut where *C. albicans* is a commensal. It is also important for utilization of non-fermentable carbon sources, some amino acids, and for regulation of hyphal growth. Both Goa1 (discussed later) and Mcu1 are possible antifungal targets, but as they are restricted to the “CTG clade” of *Candida* species, they cannot be the targets for a broad-spectrum chemical, whereas components of ERMES potentially can be.

## Mitochondrial inheritance, recombination and genotype impact the pathogenicity of populations

The mitochondrial genome can significantly impact the virulence potential of fungi. For instance, genetic crosses in the plant pathogen *Heterobasidion annosum* showed that the mitochondrial genome controls the virulence of hybrids [24]. Homokaryons and heterokaryons with mitochondria of S (less virulent on pine seedlings) and P (more virulent on pine seedlings) groups showed virulence based on the mitochondrial genotype irrespective of the nuclear genotype. Hybrids with the P type mitochondria are more virulent compared to those with the S type mitochondria.

One reason that microbes are able to cause disease is their ability to evolve faster than their hosts. A mechanism for adaptation is nuclear recombination as a consequence of either meiotic or mitotic chromosome exchanges. Few efforts have been taken to unravel the role of mitochondrial recombination in the emergence and maintenance of virulence in populations, largely because of the predominant assumption of uniparental inheritance of the mitochondrial genome. The mitochondrial genotype has been correlated with the virulence of *C. deuterogattii* (previously *C. gattii* VGII), the species responsible for almost 90 percent of cases in the cryptococcosis outbreak in areas in western Canada and northwestern United States [53,54]. Mitochondrial recombination occurs in laboratory crosses between *C. deuterogattii* strains and between *C. gattii* × *C. deuterogattii* [53]. Moreover, the physical proximity of *C. gattii* and *C. deuterogattii* in the same geographical location suggests such interspecies hybrids may arise in nature. Mitochondrial recombination has also been observed in *C. deuterogattii* × *C. tetragattii* crosses, suggesting this to be a more prevalent phenomenon than expected [54]. In a recent comprehensive study on the evolution of the *C. gattii* species complex, *de novo* genomes of 16 isolates were assembled [55]. Amongst isolates from both environmental and clinical settings that spanned four species, *C. gattii, C. deuterogattii, C. bacillisporus*, and *C. tetragattii*, there was evidence of mitochondrial recombination. For crosses between some of the isolates recombination rates were actually higher in the mitochondrial genome than in the nuclear genome [55]. Three of the *C. gattii* (VGI) isolates show higher mitochondrial sequence similarity to the *C. deuterogattii* (VGII) mitochondrial genome, and one of the isolates has a genotype more similar to *C. tetragattii*. From a cross between *C. deuterogattii MAT***a** and *C. bacillisporus MAT*α isolates, one progeny was isolated with a chimeric mitochondrial gene encoding NADH ubiquinone: half of the allele was derived from *C. bacillisporus* and other half from *C. deuterogattii*, and 12 other mitochondrial genes having genotypes similar to *C. deuterogattii* [55–56]. Remarkably, in the same strain the nuclear genome was nearly isogenic with the *C. bacillisporus* parental genotype, indicating a fascinating case of mitochondrial recombination in the absence of nuclear recombination during the sexual cycle [55].

With the predominance of the view that most of the mitochondrial inheritance in *Cryptococcus* species is uniparental, the observed recombination of mitochondrial genomes could be attributed to the *MAT*α × *MAT*α “same sex” mating process that occurs in *Cryptococcus* species [57-59]. This phenomenon of recombination is hypothesized to have contributed in the origin and spread of hypervirulent strains, with enriched virulence traits. This hypothesis is further supported by the proposed role of mitochondrial morphology in the virulence of *C. deuterogattii* [19]. Identification and characterization of genes governing this recombination process can be a major milestone, although the task is not straight forward due to the divergence of genus *Cryptococcus* from the ascomycetes in which considerably more is known about mitochondrial properties. For instance, the homologs of *S. cerevisiae* genes *MHR1* and *CCE1*, with a direct role in mitochondrial recombination, while present in *Candida* species are absent from *Cryptococcus* species.

Mitochondrial recombination is more prevalent in fungal pathogens than previously thought, and is also observed in opportunistic pathogens such as *C. albicans* and *A. fumigatus*. Although these fungi mostly propagate through the asexual mode of reproduction, and most of their populations are clonal, there is evidence of genetic recombination events in both their nuclear and mitochondrial genomes. These pathogens show “cryptic sex”, with mating type (*MAT*) loci and meiosis-associated genes. In the case of *A. fumigatus*, there is a characterized sexual cycle and crossing has been optimized to the extent that isogenic isolates have been generated by a series of backcrosses [60,61]. These isogenic mating pairs were used to show that mating type has no role in *A. fumigatus* virulence. Crosses with *A. fumigatus* isolates such as the isogenic pair and analysis of progeny may similarly test if the mitochondrial genotype has any contribution to virulence. In diploid *C. albicans*, cell fusion events between opposite mating type cells give rise to tetraploid cells, which then undergo a parasexual cycle with recombination and chromosomal loss events generating progeny of diploid and aneuploid cells [62,63]. Although nuclear recombination events are preferentially studied in *C. albicans* and other *Candida* species, mitochondrial recombination events have also been reported [64-66].

Compared to nuclear recombination events, which can also result from mitotic recombination within a diploid genome, mitochondrial recombination could be perceived as even stronger evidence of genetic exchange between different cells. A study of 45 clinical isolates of *C. albicans* and three reference strains involved sequencing seven regions of the mitochondrial genome, to look for nucleotide sequence variations for each strain [66]. This study generated data of 2553 nucleotides with 62 single nucleotide polymorphisms and seven indels (insertion or deletion of nucleotide bases), thereby defining nine mitochondrial haplotypes [66]. A single most parsimonious tree from the analysis suggested mostly clonal propagation for *C. albicans*; however, homoplasy was present (a character shared by different species but absent from common ancestors), suggesting the occurrence of mitochondrial recombination events to allow genetic exchange. Recombination events provide an opportunity for new strains of the fungus with variable virulence (as is the case of the *C. gattii* species) where in the past occasional mating events occurred. As there is clear evidence of mitochondrial recombination in *Candida* species [64-66], this suggests that they have mitochondrial recombination machinery.

The findings of mitochondrial recombination events by Anderson et al. (2001) [66] were followed by others, expanding to other pathogens of the *Candida* clade. A multi-locus sequence typing (MLST) study of 36 *C. albicans* strains from a geographically diverse population identified 66 polymorphic sites in seven sequenced regions [64]. Higher diversity in mitochondrial haplotypes was observed compared to the previous study [66], with a total of 19 haplotypes of the mitochondrial genome and evidence of recombination amongst the haplotypes in natural populations. Out of the 19 haplotypes 18 were from the set of 24 strains alone. Analysis of the “Psilosis” complex consisting of *C. parapsilosis, C. orthopsilosis*, and *C. metapsilosis*, also suggests possible events of intraspecies genetic exchange leading to mitochondrial recombination mainly in *C. orthopsilosis* [65]. Mitochondrial genomes were studied from 18 strains of the complex isolated from geographically diverse locations in Europe, South America and the USA. *C. orthopsilosis* mitochondrial genomes showed high diversity, with about 241 substitutions and more than 50 indels of length ranging up to 201 bp amongst strains of the three species and evidence for past mitochondrial recombination events. This level of variation amongst mitochondrial genomes of *Candida* species suggests there is an input from selection. As mentioned previously, recombination followed by genetic exchange can potentially give rise to more pathogenic species; nuclear genetic exchanges between two non-pathogenic parental strains gave rise to the new globally-distributed pathogen *C. metapsilosis* [67]. This landmark study involved the sequencing of 11 clinical isolates of *C. metapsilosis* from diverse geographical locations and comparing their genomes of the two parents *C. orthopsilosis* and *C. parapsilosis* to trace the evolution of the species and the genetic basis of its virulence.

For mitochondrial recombination in pathogenic *Aspergillus* species, more research is required as there is insufficient information on the mitochondrial recombination process at present. Most of the knowledge about pathogens like *A. fumigatus* and *A. flavus* is from endeavors to generate mitochondrial genome sequence information and annotation [31]. The different levels of interspecies variation in the mitochondrial genomes of six different *Aspergillus* species, and also in *Penicillium* species, suggest that mitochondrial recombination may have occurred. However, such recombination events are yet to be demonstrated. There is evidence for mitochondrial recombination events in non-pathogenic *Aspergillus* species, although these rearrangements are mostly credited to the mobile introns in the mitochondrial genome [68].

For a long time the view was that mitochondrial inheritance is uniparental in members of the *C. gattii* species complex, as reported in *C. neoformans* [69,70], where the mitochondria are predominantly inherited from only one mating type. Recent studies on mitochondrial biology of members of the *C. gattii* species complex indicates, however, that both uniparental and biparental mitochondrial inheritance exist [56,71]. The mitochondrial inheritance pattern differs between crosses in *C. gattii* species. “Out-group” crosses between species *C. gattii* (VGI) × *C. bacillisporus* (VGIII) had uniparental mitochondrial inheritance while “in-group” crosses, within *C. deuterogattii* (VGII × VGII), demonstrated biparental mitochondrial inheritance [56,71]. An informative discovery was made by comparing mitochondrial inheritance in crosses between non-isogenic parents with crosses between isogenic parents: these showed that the previous conclusion that uniparental mitochondrial inheritance in *Cryptococcus* species is controlled by the mating type locus was a consequence of the strains used [71]. Rather, multiple determinates in the nuclear genome control mitochondrial inheritance. One of these determinants is the mating type locus such that when the strains are identical except for this region, mating type becomes the sole controlling factor and that leads to uniparental inheritance of the mitochondria from the *MAT***a** parent (discussed in [72]). The *C. gattii* species show no single pattern of inheritance. However, these studies provide insight into the role of the mitochondrial genome as an independent entity for the virulence of species in the *C. gattii* complex [56,71]. Isogenic strains with different mitochondrial genotypes serve as tools, without any background complications from the nuclear genome, for studying the role of mitochondrial genotypes in virulence of *C. deuterogattii*. These ‘**a**’ and ‘α’ *C. deuterogattii* isogenic strains show equivalent pathogenicity in a mouse model, irrespective of their mitochondrial genotype [71].

Variance in mitochondrial genotype is one of the factors accounting for differences in virulence in *C. gattii* species [19]. Voelz et al. (2013) showed that in the progeny of out-group (interspecies) crosses there is an association between intracellular proliferation rate (IPR), mitochondrial tubularization and mitochondrial donor phenotype [56]. That is, no enhanced tubularization or IPR was associated with the progeny that have the *MAT***a** type of mitochondria from a low virulence strain. However in the opposite cross, where the *MAT***a** parent mitochondria are from the hypervirulent strain, this association of IPR and tubularization was variable, with IPR and mitochondrial tubularization phenotype ranging from very high to very low and even surpassing the original parental phenotypes. For the “within species” *C. deuterogattii* crosses, the mitochondria in the hypervirulent strains can be inherited from either the **a** or α parent, but progeny maintain the mitochondrial tubularization phenotype.

**Figure 1.**
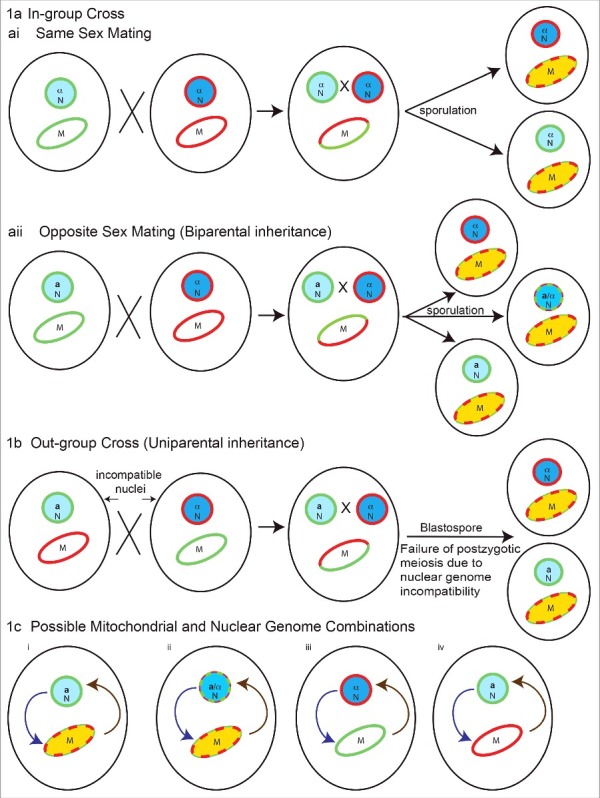
Possible nuclear and mitochondrial genetic genotypes in *Cryptococcus* species in progeny from crosses contributing to their virulence. 1a) In-group cross (within species), two possible scenarios: (ai) same sex mating (α-α), recombinant mitochondrial genomes, with isogenic nuclei, and (aii) opposite sex mating (**a**-α) with both recombinant mitochondrial and nuclear genomes generated in progeny. In these crosses mitochondrial inheritance is biparental, with contribution from single parent at a time. The exception is crosses between isogenic strains, where mitochondrial inheritance is uniparental. 1b) Outgroup cross (interspecies): the nuclear genome can be genetically incompatible leading to post-zygotic meiotic failure, but retaining the possibility of recombination amongst mitochondrial genomes. In such a scenario progeny will have recombinant mitochondria with nuclei isogenic to one of the parents. 1c) Possible mitochondrial and nuclear genome combinations: (i) recombinant mitochondrial genome in isogenic nuclear background, (ii) recombinant mitochondrial genome in a recombinant nuclear background, (iii) non-recombinant mitochondria from first parent with non-recombinant nuclei from another parent and (iv) vice versa of iii. Interorganelle (nuclear-mitochondrial) regulatory loops on each other are shown. Light blue circles with green outline and dark blue circles with red outline represents nuclei with different genetic background respectively. Blue circles with mixed dotted outline represents recombinant nuclei. The **a** and α represents mating types. Ellipse with green and red outline represents mitochondria with different genetic background respectively. Ellipse with red green dotted outline and yellow color represents recombinant mitochondria. Curved arrows in 1c represents interorganelle regulatory signaling loops for nucleus to mitochondria (purple) and mitochondria to nucleus (brown).

These genetic studies indicate that the mechanisms by which mitochondria contribute to *C. gattii* species virulence are not simple and reflect an interplay between the mitochondrial genotype and nuclear genotype. The contribution of mitochondrial inheritance and recombination in promoting virulence depends on the genetic background and crossing scenario, as illustrated in Fig. 1. From Fig. 1a and 1b, four possible combinations of nuclear and mitochondrial genomes can be generated as shown in Fig. 1c. First is a recombinant mitochondrial genome in an isogenic nuclear background, the second scenario is a recombinant mitochondrial genome in a recombinant nuclear background. The third scenario is non-recombinant mitochondria from the first parent with non-recombinant nuclei from the second parent, and the fourth scenario is opposite of the third, with non-recombinant mitochondria from the second parent and non-recombinant nuclei from the first parent. In all four different combinations, due to differences in the mitochondrial and nuclear genetic makeup of progeny, interorganelle (nuclear-mitochondrial) regulatory loops can differ and impact the virulence of progeny. As such, the contribution of the mitochondrial genotype to virulence requires further investigation, not as an isolated organelle, but rather in the context of interactions or signaling of mitochondria with other major organelles, particularly the nucleus and endoplasmic reticulum. Evidence for the species in *C. gattii sensu lato* suggests that the full expression of virulence traits and proper mitochondrial morphology in virulent strains requires a compatible nuclear genotype.

## Mitochondrial-nuclear signaling and its link to drug responses, fitness and pathogenesis

**Figure 2.**
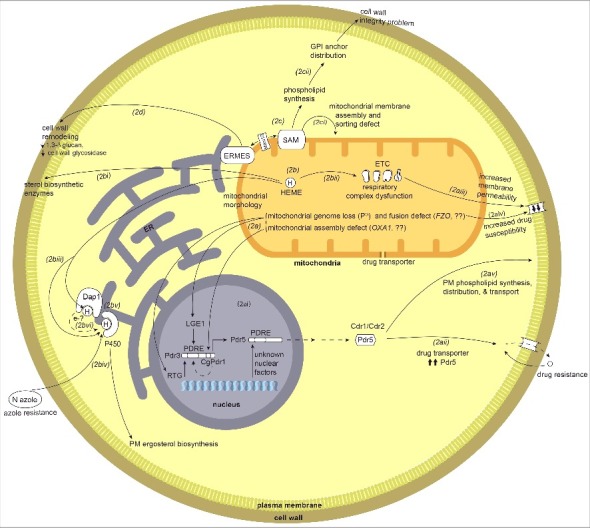
Overview of mitochondrial crosstalk with other organelles and cellular components such as the nucleus, endoplasmic reticulum (ER), the sorting and assembly machinery (SAM), plasma membrane (PM), and cell wall. 2a) Mitochondrial dysfunction or defects triggers mitochondrial-nuclear retrograde signaling. Loss of mitochondrial genome and mitochondrial fusion defect via unknown mechanisms activates nuclear retrograde (RTG) genes. 2ai) The mitochondria-nuclear retrograde signaling genes have role in regulating expression of *PDR* genes. Change in *RTG* gene expression in turn affect the expression of *PDR3*. Pdr3/CgPdr1 has an auto-regulatory loop (dotted curved arrow inside nucleus) and RTG functions to regulate Pdr3 expression. Pdr3/CgPdr1 and other unknown nuclear factors regulates expression of drug transporter Pdr5/CDR1/CDR2 by acting on its PDRE. One of the factors is Lge1, a nuclear protein downstream of Psd1, as ρ° cells increase the expression of Pdr3 and Pdr5. 2aii) Increase in the expression of drug transporter Pdr5 leads to increase in drug efflux hence drug resistance. 2aiii) Mutation and dysfunction of mitochondrial respiratory complex perturbs overall cells energy state and also possibly affects the membrane fluidity (perturbed ergosterol biosynthesis), which in turn leads to down regulation of drug transporters *CDR1*/*CDR2* and hence increased drug susceptibility. 2aiv) Mitochondrial dysfunction, such as a fusion defect or loss of the mitochondrial genomes affects lipid biosynthesis and can in turn perturb the membrane permeability. 2av) Drug transporters Cdr1/Cdr2/Pdr5 have a role in synthesis of phospholipids, their distribution and transport across the plasma membrane. 2b) Mitochondria are the site for heme biosynthesis and at the center of regulating cellular heme homeostasis and its link to azole drug resistance. (2bi-2biii) Heme is the cofactor for enzymes involved in sterol biosynthesis enzymes, as a cofactor for mitochondrially-located respiratory complex enzymes and ER-localized cytochrome P450. 2biv) The heme-binding catalytic site for cytP450 can also be bound by azole drugs, and hence when targeted can impair ergosterol biosynthesis. 2bv) Heme-binding protein, Dap1, co-localizes with cytP450 at the ER. 2bvi) Dap1 dependent stabilization of cytP450, possibly by channeling of electrons from Dap1 heme to cytP450 heme. 2c) Role of mitochondrial outer membrane complex SAM (sorting and assembly machinery) in maintaining cell wall integrity and mitochondrial outer membrane function. 2ci) Dysfunction of SAM components impact mitochondrial membrane assembly and sorting of proteins through mitochondrial membranes and matrix. 2cii) Mutations or dysfunction of SAM core components leads to phospholipid synthesis problems which in turn affects the distribution of glycosylphosphatidylinositol (GPI) anchor protein and lead to a reduction in cell wall integrity. 2d) Involvement of ERMES (endoplasmic reticulum-mitochondria encounter structure) and the mitochondrial-ER contact site in maintaining mitochondrial morphology. ERMES mutants have perturbed cell wall remodeling with decreased expression of cell wall glycosidase *PHR1* and 1,3-β-glucan affecting cell wall integrity.

Communication and regulation through mitochondria to the nucleus is termed mitochondrial retrograde signaling. The phenomenon was discovered in the model yeast *S. cerevisiae* [73], where two important mitochondrial-nuclear signaling genes *RTG1* and *RTG2* were identified [74] (although these genes are not universally conserved in fungi as assessed by BLAST against the NCBI and fungal-specific genome databases). Retrograde signaling in *S. cerevisiae* has been primarily studied in the context of altered nutrient metabolism in response to changes in mitochondrial function. However, there are many other important aspects associated with retrograde signaling. A genetic screen for the negative regulators of the pleiotropic drug resistance genes (PDR) and a microarray-based genome-wide transcriptional study of mitochondrial dysfunction revealed that in *S. cerevisiae* cells lacking the mitochondrial genome (ρ° cells) there is strong induction of the *PDR* genes (Fig. 2a) including *PDR5*, which encodes an ATP-binding cassette (ABC) transporter [75-77]. *PDR5* transcriptional regulation is under the control of Zn(II)_2_Cys_6_ transcriptional regulator proteins Pdr1 and Pdr3. Increased drug resistance and up-regulation of *PDR5* expression in case of mitochondrial dysfunction is regulated by Pdr3 but not Pdr1. Increased *PDR5* expression, with Pdr3 binding to the Pdr1p/Pdr3p response element (PDRE) of *PDR5*, is through post-translational stabilization or activation of the transcription factor [75]. Loss of the mitochondrial genome can lead to activation of *RTG* genes and hence the retrograde signaling pathway (Fig. 2a) [74]. Also, absence of *RTG* genes decreases the expression of *PDR3*, possibly through inactivation of a Pdr3 autoregulation loop involving these retrograde signaling genes (Fig. 2ai). Nuclear-mitochondrial signaling becomes particularly important when the cell's mitochondria are under stress; as illustrated in humans where this is associated with the onset of many “mitochondrial” diseases [78].

Increased expression of *PDR5* was also observed as a consequence of the loss of genes involved in mitochondrial fusion. These are the *FZO1* gene, required for fusion of mitochondrial outer membranes, and the *OXA1* gene, which encodes a mitochondrial protein required for the assembly of the cytochrome c oxidase complex and the F_0_ ATPase sub-complex, both of which are inner mitochondrial membrane complexes [75,79], (Fig. 2a). In *fzo1* mutant cells *RTG* gene expression is required for up-regulation of Pdr3. Hence, mitochondrial dysfunction can initiate mitochondrial-nuclear crosstalk to contribute to drug resistance. Looking at the intricacy of this signaling loop (Fig. 2a) as defined through research on *S. cerevisiae*, the connection of drug resistance to mitochondrial retrograde signaling is an interesting avenue to be explored in pathogenic fungi, especially with the increasing problems of fungicide resistant strains.

The findings from *S. cerevisiae* have been extended to *Candida* species, where respiratory loss or dysfunction of the mitochondrial genome is often associated with increased resistance to azole drugs (reviewed in [80]). The drug resistance phenomenon reported in *S. cerevisiae* has parallels in *C. glabrata* where transcription factor *PDR3* homolog *CgPDR1*, upon compromised mitochondrial function, upregulates the expression of efflux pump encoding genes *CDR1* and *CDR2*, the homologs of *S. cerevisiae* ABC transporter gene *PDR5* (Fig. 2ai) [81-84]. Increased drug resistance in *C. glabrata* correlates with increased virulence and fitness of the strains expressing mutated *CgPDR1* via up regulation of the efflux pump activity [85] (Fig. 2aii). Also, clinical isolates show higher levels of drug resistance that correlates with mitochondrial dysfunction, which is further verified in a mouse model of disease [83,86]. In *C. glabrata* loss of the mitochondrial genome leads to increased drug resistance; however, in other species of *Candida*, such as *C. albicans*, this phenomenon is more difficult to assess as they are obligate aerobes and do not survive the loss of their mitochondrial genomes (i.e. they are petite negative).

Mitochondrial genome loss is a key trigger for the activation of the Pdr pathway and hence drug resistance, but is not the sole trigger [87,88]. Other than mitochondrial genome loss there are additional signaling pathways merging on Pdr activation, which become more important specifically in obligate aerobes such as some *Candida* and *Cryptococcus* species. After reviewing the research, Shingu-Vazquez and Traven proposed a compelling argument that a key signal is altered lipid homeostasis [80]. Pdr genes are involved in drug resistance and also in restoring membrane lipid homeostasis through the roles of proteins such as Psd1 and Pgs1 and others involved in respiratory homeostasis [80]. Also in *C. albicans* there is evidence that mutations of the mitochondrial respiratory complex genes lead to down regulation of drug transporter genes, which leads to increased drug susceptibility [21] (Fig. 2aiii). Hence, there is a connection between regulation of drug transport and mitochondrial function.

The relationship between drug resistance and mitochondrial dysfunction involves several different genes in *Candida* species. Two of the genes are *PSD1* (encoding phosphatidyl serine decarboxylase) [82,89], and *PGS1* (encoding phosphatidylglycerolphosphate synthase) [90]. Mainly involved in membrane lipid biosynthesis, these genes also have roles in regulating drug resistance. Overexpression of *PSD1* in *S. cerevisiae* affects the Pdr3-dependent *PDR5* expression and drug resistance via a regulatory loop involving nuclear protein Lge1 [89], and this regulatory loop seems partly to have its parallels in *C. glabrata* [82]. The *CgPGS1* deletion strain has a decrease in drug susceptibility likely due to increased levels of transcripts of drug efflux transporters [90]. The compromised state of phospholipids in the plasma membrane or mitochondrial membrane and/or changes in cell wall integrity are suggested to influence resistance to drugs [91] (Fig. 2aiv). Along with the up-regulation of the drug transporter, *PDR* genes are also involved in phospholipid and membrane protein synthesis. Many of the drug transporters, such as Cdr1, Cdr2, Pdr5 or Yor1, are involved in phospholipid distribution and transport across membranes [92] (Fig. 2av); hence the relationship between mitochondrial/cellular membrane dysfunction and drug resistance includes another level of complexity. As some of these drug transporters are a constitutive part of the mitochondrial membrane but are encoded by the nuclear genome, membrane abnormality or mitochondrial stress can be transduced to the nuclear factors or yet unknown regulators (Fig. 2a & 2ai). These nuclear proteins in turn regulate the synthesis of transporter genes to restore membrane homeostasis and at the same time influence the levels of drug susceptibility in fungal pathogens.

There are also links between azole resistance and mitochondrial function in pathogen *A. fumigatus* [38,93]. In *A. fumigatus* mitochondrial fission mutants have increased resistance to voriconazole. Repression of lanosterol 14 α-demethylases (e.g. Cyp51A), the azole drug target (which will be discussed in more detail later), leads to an azole susceptibility phenotype. However, deleting fission gene *DNM1* in a Cyp51A tet-on repressive strain reverses this susceptibility to increase azole resistance, linking mitochondria to ergosterol biosynthesis and drug resistance in *A. fumigatus* [38]. Another link of mitochondria to azole drug resistance is through the mitochondrial complex I [93]. Deletion of genes pertaining to this complex have widespread effects including misregulation of gene clusters for secondary metabolite biosynthesis, increased azole resistance and decreased virulence in an immunocompromised murine model. The azole resistance observed in complex I gene deletion mutants is independent of increased ergosterol levels or expression levels of the drug targets *CYP51A* and *CYP51B*, as the levels for these genes were comparable to wild type levels. However, the azole drug resistance in *A. fumigatus* in complex I mutant strains is opposite of what is observed for *C. albicans* where loss of complex I subunits leads to increased azole susceptibility [21].

We have primarily focused on drug resistance caused by mitochondrial dysfunction, but the story is not straightforward. While a limited set of mitochondrial dysfunctional states or specific mutations induce azole drug resistance, for a few they cause increased drug susceptibility, even with the increased expression of efflux pumps [94]. There are examples in *Candida* and *Cryptococcus* species and other pathogenic fungi where mitochondrial dysfunction, either due to membrane permeability defects or increased sensitivity to oxidative stress, causes respiratory malfunction and thereby leads to susceptibility to antimicrobial drugs other than azoles (Fig. 2aiii). For example, the *C. albicans fzo1*Δ strain has fusion-deficient mitochondria and has increased azole susceptibility due to a decrease in the efflux activity (Fig. 2aiv) of Cdr1 because of its missorting to the vacuole and also perturbed levels of phospholipids [42]. A second example is *C. albicans* where deletion mutants of the mitochondrial respiratory complex related genes (*goa1*Δ and *ndh51*Δ) show down regulation of drug transporter genes, which leads to increased drug susceptibility (Fig. 2aiii). Both mutants have a compromised cellular energy state. The *goa1*Δ mutant has more extensive defects in expression of genes encoding peroxisomal components and gluconeogenesis, and a decrease in expression of genes encoding mitochondrial carrier proteins, such as *TIM22* and *YMC2*, and ergosterol biosynthesis genes. The *ndh51*Δ mutant also has down-regulation of ergosterol biosynthesis genes [21].

Microarray-based transcriptional studies on strains with dysfunctional mitochondria in *S. cerevisiae* yielded significant information on inter-organelle cellular signaling responses under stress [77,95]. These studies included transcriptional profiling of respiratory deficient petite strains, strains lacking *RTG* genes, and strains treated with different oxidative phosphorylation inhibitors. Insights were gained into cellular pathway changes by observed differential expression of genes related to metabolic remodeling, peroxisome biogenesis, retrograde signaling dependent genes such as some TCA cycle and propionate metabolism genes, glycolytic genes, cellular membrane transporter genes (involved in pleotropic drug resistance phenotype, *PDR*), genes related to sporulation, cell wall integrity pathway, and genes regulated by signaling systems such as the cAMP-PKA and Hap pathways. However, equivalent transcript profiling has not yet been done in pathogenic fungi.

## The essential link between iron-heme and the mitochondria

Mitochondrial dysfunction has a broad impact on azole drug resistance because the mitochondrion is the site for synthesis of heme, an important enzyme cofactor [2] (Fig. 2b). As heme is the cofactor for enzymes involved in sterol biosynthesis (Fig. 2bi), respiratory chain enzymes (Fig. 2bii) and cytochrome P450 enzymes (Fig. 2biii) [96], any change in cellular levels of heme directly impacts the function of such enzymes. Heme is a cofactor for the ER-localized class II P450 monooxygenase Cyp51/Erg11, the lanosterol 14 α-demethylase that catalyzes a key step in ergosterol biosynthesis [97]. This particular P450 is of interest as it is the target for antifungal azole drugs such as fluconazole, ketoconazole or vorizonazole. The azole drugs interact with the heme-binding catalytic site of the enzyme [98] to impair the synthesis of ergosterol (Fig. 2biv). Evolved resistance to these antifungals is a pressing problem in medical mycology, and one mechanism for resistance is the reduced affinity of these drugs to Cyp51 [99]. Mutations in the *CYP51* gene include changes in the protein's heme binding site, G464S and R467K, that reduce affinity to fluconazole.

Any changes in the cellular heme homeostasis can lead to increases in azole resistance. Heme levels govern the intricate balance of azole resistance to susceptibility in pathogenic *Candida* and *Aspergillus* species, an added regulation of P450 activity [100,101]. Dap1 (damage resistance protein 1), which contains a heme-binding domain, is at the center of this heme-based P450 regulation, as reported originally in *S. cerevisiae* [102,103]. Subsequent experiments in *Candida* and *Aspergillus* species further reinstates the heme-dependent Dap1-regulated activity on Erg11 and its link to azole drug resistance. In *A. fumigatus* there are three homologs for Dap1, *viz* DapA, DapB and DapC, and they all link to heme-dependent P450 regulation [101]. DapA colocalizes with Erg11 in the ER (Fig. 2bv) and in a heme-dependent manner stabilizes Erg11 post transcriptionally and hence contributes to azole drug resistance in the pathogen. One possibility is that Erg11 is stabilized through channeling electrons from Dap1 heme to P450 heme (Fig. 2bvi). Loss of DapA or mutation of its heme-binding site perturbs ergosterol synthesis and makes the fungus more susceptible to azole drugs. These phenotypes can be rescued by adding heme extracellularly to the growth medium, showing that heme is an important regulatory factor. On the other hand, DapB and DapC act as negative regulators of P450, in both heme-dependent and independent mechanisms, as both of these proteins make the fungus more susceptible to azoles, with DapC seeming to be a more potent effector of the two. A similar role of Dap1 occurs in *C. glabrata*, where the protein, in a heme-dependent manner, is required for proper functioning of Erg11 and provides the fungus with increased azole tolerance [100].

The *A. fumigatus* iron transporter MrsA, a homolog of *S. cerevisiae* high affinity iron transporter Mrs4, is another important contributor in maintaining cellular iron homeostasis [104]. This mitochondrial iron transporter plays an important role in regulating cellular iron homeostasis by affecting the expression of transcription factor, SreA, which is involved in maintaining cellular iron homeostasis by repressing expression of iron assimilator genes under high cellular iron conditions [104,105]. A deletion mutant of *mrsA* has lower mRNA levels of *sreA* and higher expression levels of reductive iron assimilation system genes *ftrA* (ferroxidase), *fetC* (iron permease) and siderophore-mediated iron acquisition system genes *sidA* (catalyzes siderophore synthesis) and *mirB* (siderophore transporter) all of which are repressed by SreA. On the other hand, genes pertaining to iron consumption such as cytochrome C are repressed in this mutant, probably an attempt to restore cellular iron homeostasis. Higher oxidative stress levels and increased azole susceptibility are observed in *mrsA* deletion mutants, both of which are reversed in the presence of reactive oxygen species (ROS) scavenger l-ascorbic acid. This cellular dysfunctional state due to absence of MrsA is translated in decreased virulence of the *mrsA* deletion strain in comparison to parent wild type and *mrsA* complemented strain in an immunocompromised murine infection model. MrsA is an example that emphasizes the significance of mitochondrially-targeted proteins in deciding the outcome of a host-pathogen interaction.

Iron and heme are important constituents of mitochondrial enzymes (Fe-S clusters, biogenesis reviewed extensively in [106,107]) and have clear role in azole drug resistance. Extending the role of heme and iron, one of the key protein complexes involved in the regulation of mitochondrial functions is the HAP complex [108]. This complex is heme-activated, glucose-repressed, and is a global regulator of respiratory gene expression. In *S. cerevisiae* Hap2/3/5p (CBC) forms the core DNA binding domain that targets CCAAT sites, and Hap4 is the activating subunit [109]. Hap core subunit orthologs are conserved in other fungal species, including pathogens. In *Aspergillus*, the ortholog of Hap2, HapB interacts with HapX (bZIP protein) [110] by the conserved N-terminal CBC-interacting domain of activator subunit Hap4p, and is involved in iron dependent mitochondrial gene regulation and is a virulence factor [111,112]. In *A. fumigatus* HapX is essential for adapting to iron starvation, and the *hapX* deletion mutant has attenuated virulence [111]. HapX forms a regulatory loop with SreA: under iron depleted conditions HapX represses the transcription of genes involved in iron consumption as well as *sreA* expression, while under iron replete conditions SreA represses expression of genes required for iron uptake and *hapX*. Almost 31% of genes are repressed by HapX, including those for the TCA cycle, Fe-S cluster biosynthesis, respiration, and encoding amino acid metabolism proteins localized to mitochondria, and under HapX deficiency there is an increase in mitochondrial DNA content. This emphasizes the significance of HapX in maintaining mitochondrial health and metabolism. HapX also affects heme biosynthesis, as can be seen from accumulation of the heme precursor protoporphyrin IX in the *hapX* deletion mutant during iron starvation conditions.

HapX and its iron dependent regulatory roles are also present and impact virulence in pathogens in the *Cryptococcus* and *Candida* genera [113,114], and in plant pathogens such as *Fusarium oxysporum* [115]. In *Cryptococcus*, HapX negatively regulates respiratory and TCA cycle genes under iron deplete conditions and positively regulates iron uptake genes, such as a siderophore transporter Sit1 and GATA factor Cir1, which is a key regulator of virulence factors [114]. HapX and Hap3 mutants have poor growth on hemin (oxidized heme) as the sole iron source, possibly due to a defect in uptake or utilization, and have reduced transcript levels of genes encoding ergosterol biosynthesis enzymes when grown under low iron conditions. This established a possible role of Hap proteins in azole drug sensitivity, given that heme is a cofactor for the cytochrome P450 azole drug target. In *C. neoformans* much of the HapX related mitochondrial function regulation is also attributed to Mig1, a zinc finger protein [116]. Mig1 positively regulates *HAPX* transcription under low iron conditions, and regulates expression of a subset of the HapX-regulated genes such as those encoding aconitases (which are Fe-S containing enzymes), heme biosynthetic enzymes, and proteins involved in iron acquisition. Also, *MIG1* transcript levels increase under both iron-depleted and iron-replete conditions in a *HapX* deletion mutant, suggesting functional interaction between two proteins. *Mig1* deletion mutants have increased azole drug susceptibility that can be possibly explained as Mig1 regulates the expression of heme biosynthetic enzymes and, as stated previously, heme is an essential cofactor for cytochrome P450s. The HapX homolog, Hap43, is conserved in *C. albicans*, and has roles in the repression of iron consuming mitochondrial genes, including heme biosynthetic genes, and the deletion strain has attenuated virulence in murine infection model [113].

A key biochemical function that occurs in mitochondria is the synthesis of Fe-S clusters. Atm1 transports Fe-S precursors from mitochondria into the cytosol. Recently, this mitochondrially-localized transporter has been identified in *C. neoformans* from a role in responding to high concentrations of copper ions [117]. Atm1 likely has a role in virulence, although this is difficult to assess since the gene appears to be essential, and is based on reduced proliferation within macrophages of a strain with altered regulation of *ATM1*.

## Mitochondrial impacts on other cellular compartments

In addition to membrane and sterol impacts, mitochondria also contribute to cell wall integrity. Compromised functioning of a protein complex associated with the mitochondrial outer membrane, abbreviated SAM for sorting and assembly machinery, in *C. albicans* affects cell wall integrity, protein distribution and homeostasis (Fig. 2c) [118]. The SAM complex is well characterized in *S. cerevisiae* with core components of the complex being Sam50 (a β-barrel channel forming protein), Sam35 (β-signal binding protein) and Sam37 (aids in release of bound precursor protein), with Sam50 and Sam35 as essential components for viability [119,120]. Other transient or shared members of the complex are Mdm10 (also tethered with ERMES [121]) and Mim1. This complex's main function is assembly of β-barrel-containing proteins and in conjunction with TOM (translocase of outer membrane) plays an essential role in sorting and transfer of proteins within mitochondrial membranes and into the mitochondrial matrix, and also in phospholipid homeostasis.

*C. albicans* has a homologous SAM complex, with both similarities and differences compared to *S. cerevisiae* [118,122], [123]. In *C. albicans* a *sam37* mutant has substantial fitness defects relative to the *S. cerevisiae sam37* mutant, and also loss of the mitochondrial genome, which is not observed in *S. cerevisiae*. Both *S. cerevisiae* and *C. albicans sam37* mutants have abnormal mitochondrial morphology [118,122]. The *S. cerevisiae sam37* mutant has impaired phospholipid homeostasis, a phenotype with possible implications in *C. albicans*, where the loss of Sam37 affects the cell wall integrity and virulence [118,122]. Mutant strains are highly sensitive to cell wall targeting compounds such as caspofungin, calcofluor white and congo red, and inhibited by low concentrations of azole drug fluconazole, and have a virulence defect in the systemic candidiasis murine model of infection [118]. The defects in the cell wall of *sam37* mutants of *C. albicans* can possibly be attributed to a defect in phosphatidylethanolamine (PE) biosynthesis that ultimately affects the glycosylphosphatidylinositol (GPI) anchor protein distribution in the cell wall, challenging its integrity (Fig. 2cii). *C. albicans* has an additional member in the SAM complex, annotated as Sam51 that has a role in β-barrel assembly, and is absent from *S. cerevisiae* [123]. The complex has also assembly differences, such as in *S. cerevisiae* Sam37 is essential for the assembly of VDAC, an outer membrane voltage dependent anion selective channel required for metabolite exchange between cytosol and mitochondria; however, the *C. albicans sam37* mutant has only a mild impairment in VDAC assembly. Also the SAM dependent assembly process of the TOM complex is much faster in *C. albicans* compared to the kinetics in *S. cerevisiae* [123].

An impact of mitochondrial dysfunction is associated with the unfolded protein response in the endoplasmic reticulum (ER). Misregulated or disrupted mitochondrial morphology affects mitochondrial-ER communication [124,125]. The ER is the organelle where misfolded proteins are tagged and processed for execution and ERMES (endoplasmic reticulum-mitochondria encounter structure) is a complex that functions to tether mitochondria and ER [126]. The complex is of interest because components found in fungi do not have homologs in mammals.

In *C. albicans*, ERMES is associated with immune cell evasion and inflammasome activation [127]. The *C. albicans* ERMES *mmm1* mutant shows delayed NLRP3 inflammasome response in a macrophage-*Candida* model of infection, with decreased ability of macrophage lysis and hence changing the outcome of the infection [127]. Because of destabilization of mitochondrial contact sites, this ERMES mutant has a defect in mitochondrial morphology, with more clumped and globular forms instead of the tubular morphology seen in wild type cells. It is known that misregulated mitochondrial morphology and function compromises the cell wall integrity in *C. albicans* [122] (Fig. 2d). In the *mmm1* mutant there are comparatively shorter hyphae both during macrophage infection as well as in vitro. There is a decrease in expression of hyphal-specific genes and also perturbed cell wall remodeling with decreased level of cell surface 1,3-β-glucan and decreased expression of cell wall glycosidase *PHR1* (Fig. 2d). These changes in hyphal length and cell wall integrity can in turn perturb the signaling threshold required for the inflammasome activation, and the subsequent outcome of the macrophage-*Candida* interaction. The above study [127] is an example of how important maintenance of mitochondrial morphology can be in the context of mitochondrial interactions with other cellular organelles. Mmm1 is also required for virulence of *C. albicans* [128]. Hence, both the peri-mitochondrial complexes SAM and ERMES are required for the virulence of *C. albicans* [118,122,128]. These complexes, especially ERMES, are important from the perspective of pathobiology likely because of their role in the maintenance of the mitochondrial genome, in lipid homeostasis, mitochondrial protein import and mitochondrial dynamics, all of which directly or indirectly influence cell fitness [129,130].

*A. fumigatus* is another fungus where ERMES subunits have been studied, and a *mmm1* conditional mutant has a virulence defect in a *G. mellonella* model [131]. In *A. fumigatus*, strains with mutations in the ERMES component genes *mdm10, mdm12, mdm34* or *mmm1* have morphological defects to their mitochondria such that they feature giant, amorphous and occasionally interconnected organelles and aggregated nucleoids. These are distinctive differences compared to the tubular mitochondrial morphology seen in wild type strain.

## Exciting directions yet to be explored to understand how mitochondrial functionsimpact virulence

Mitochondrial genome inheritance, morphology, signaling and functions are clearly important for the virulence and sustenance of pathogenic fungi. While many directions could be pursued in the future for developing a comprehensive understanding of these phenomenon, four topics stand out as the most pressing. These are: (1) Mitochondrial genome recombination and inheritance, (2) interaction between mitochondrial and nuclear genomes, (3) mitochondrial signaling with other cellular organelles, and (4) proteins or metabolites involved in virulence that are targeted to the mitochondria. Exploring these four topics in detail will help in developing an understanding of how mitochondria are involved in virulence.

With the genome rearranging powers of recombination, capable of developing a new pathogenic species [67], it is important to elucidate the mechanism of mitochondrial recombination and the link between mitochondrial and nuclear genotypes. With increasing evidence of the mitochondrial genotype and mitochondrial recombination playing a crucial role in virulence in species of the *C. gattii* complex [53-55,^57^], there is a rising need to understand the phenomenon of mitochondrial recombination. In many fungal pathogens that have “historically asexual” life styles, such as species of *Candida* and *Aspergillus*, either there is concrete evidence of mitochondrial recombination [64-66] or there is apparent diversity in mitochondrial genotypes between strains [31], all pointing towards the existence of a mitochondrial recombination machinery. For none of the pathogens is the process understood, thus the necessity to characterize how mitochondrial genome recombination occurs. Also, which alleles in mitochondrial genomes contribute to virulence should be established. At present the mitochondrial genomes of pathogenic fungi are not able to be specifically modified. In future, if technologies such as mitochondrial CRISPR-Cas9 [132] are developed for these species it will be worth making mutations in the mitochondrial genome to uncover how specific regions contribute to virulence.

The mitochondrion does not function as an independent entity, and in the cell it constantly communicates with the nucleus and other organelles (Fig. 2). Mitochondrial-nuclear crosstalk has important implications in drug resistance [75]. Furthermore, at the population level there are nuclear genes in *Cryptococcus* species that encode proteins targeted to the mitochondria (such as *UVE1*) with a role in genome maintenance that likely contribute to the organism's capability to adapt and survive in harsh environments [133,134]. Investigating the mitochondrial-nuclear retrograde signaling in pathogenic fungi will help in deciphering such pathways and the roles they play in the functions of the nucleus or mitochondrion, or both. One clear experimental direction would be to analyze the transcriptional profiles when such major players are impaired in the pathogen, to determine how nuclear genome transcription pattern is affected under such conditions.

Proteins that control mitochondrial morphology, such as through fission-fusion, and SAM and ERMES components are important in interorganelle crosstalk, and impact the virulence and fitness of pathogenic fungi [25,42,75,118,127]. There is need to further explore mitochondrial signaling with cellular factors such as ERMES, SAM and the cell wall, to clearly understand how that contributes to these fitness defects and/or virulence changes. Knockouts for genes regulating mitochondrial fusion process in fungal pathogens, like *FZO1* for *C. albicans* and *A. fumigatus*, have phenotypes associated with azole drug susceptibility and virulence [38,42]. The fission mutants in *A. fumigatus* are important for growth and drug susceptibility [38]. Creating their knockouts in *C. deuterogattii* backgrounds, where tubular morphology is correlated with virulence, and then looking for the changes in the virulence patterns and drug susceptibility may yield insights into the virulence mechanisms of the strains that emerged as problems in a healthy human population. The function of the SAM complex in pathogenic fungi outside the *Candida* genus is unexplored. However, based on the phenotypes of mutants it is known that there are considerable differences between mitochondria or mitochondrial membrane related protein complexes, such as the ERMES complex, in comparison to ascomycete species [135]. Even within a phylum, there are major differences in SAM components and their function in *S. cerevisiae* and *C. albicans* [123]. Hence, due to the existing differences between the functionality and essentiality of the respiratory complex components and other mitochondrial proteins between different fungi such as *S. cerevisiae, S. pombe, Candida* spp. and *Cryptococcus* spp., it is possible that the function of the homologous protein may be as important or even unrelated to survival or pathogenesis of different fungi. Although mitochondria are conserved and fundamental components of eukaryotic cells, it is unwise to draw generalizations from studies performed in a small number of often distantly related species.

The mitochondria are organelles implicated in aging in mammalian systems, and similar links have been established in fungi as they age. Investigation at the molecular level has begun to reveal which factors of mitochondrial function are important for this process. For instance, the mitochondrial fission protein Dnm1 is also well studied for its role in aging and apoptosis in non-pathogen *Podospora anserina* [136,137]. At present, little is known about how aging of human pathogenic fungi may impact on the virulence of fungal populations during disease development. However, in both *C. neoformans* and *C. glabrata* the age of the cells is important for different aspects of virulence [138,139].

Using bioinformatics, predictions can be made about the localization of nuclear encoded genes. For instance, we used PSORT II [140] and MitoProt [141] on the predicted amino acid sequences of proteins of *C. albicans* that are required for virulence (Table 1). More than 70 proteins potentially have some level of mitochondrial localization, of which about 30 have >75% predicted mitochondrial localization by one or the other prediction tools, and the others are predicted to be targeted to the mitochondrion as well as nucleus or another subcellular compartment. 16 proteins are predicted to be mitochondrial by both bioinformatics methods (Table 1). The identification of known mitochondrial proteins like Goa1 [22] served as internal controls. The caveat of such bioinformatic approaches is that they may miss mitochondrial proteins: for instance, Mcu1, required for the utilization of *N*-acetylglucosamine and virulence, which is clearly mitochondrially-localized [52], is not predicted to be mitochondrial.

**Table 1.** Predicted mitochondrial proteins of *Candida albicans* that are involved in virulence. Genes listed in the Candida Genome Database [150] (http://www.candidagenome.org/cgi-bin/phenotype/phenotype.pl?observable = virulence) with a role in virulence of *C. albicans* were used for prediction of mitochondrial localization. Genes encoding proteins with localization scores ≥ 50% by MitoProt II-v1.101 and/or PSORT II are listed.

Gene	Mitoprot (probability)	PSORT II (%)	Virulence	Model	Reference	Function
*BEM1*	0.98	69.6	Decreased	mouse intravenous infection	[142]	Protein required for wild-type budding, hyphal growth, and virulence in a mouse systemic infection
*BIO2*	0.88	69.6	Absent	mouse intravenous infection	[128]	Putative biotin synthase
*BRG1*	0.87	60.9	Decreased	mouse intravenous infection	[143]	Transcription factor; recruits Hda1 to hypha-specific promoters
*GOA1*	0.9123	87	Decreased	mouse intravenous infection	[22]	Protein required for respiratory growth, resistance to oxidants, chlamydospore formation, hyphal growth, and virulence; osmotic stress
*ILV2*	0.9812	95.7	Decreased	mouse intravenous infection	[144]	Putative acetolactate synthase, regulated by Gcn4p
*LYS4*	0.8317	73.9	Decreased	mouse intravenous infection	[145]	Homoaconitase; regulated by Gcn4, Gcn2
*MGM101*	0.9849	82.6	Decreased	mouse intravenous infection	[128]	Putative mitochondrial genome maintenance protein; fungal-specific (no human or murine homolog)
*RPF2*	0.9537	52.2	Decreased	mouse intravenous infection	[128]	Putative pre-rRNA processing protein
*SFL1*	0.9628	69.6	Decreased	mouse intravenous infection	[146]	Transcription factor involved in negative regulation of morphogenesis, flocculation and virulence; induced in core caspofungin response
*SUV3*	0.9939	91.3	Decreased	mouse corneal infection	[147]	RNA helicase; mitochondrial RNA catabolism; required for chlamydospore formation, embedded hyphal growth, wild-type respiratory growth
*TIM44*	0.9526	65.2	Absent	mouse intravenous infection	[128]	Protein involved in transport across membranes
*TIM50*	0.9919	82.6	Absent	mouse intravenous infection	[128]	Predicted component of the translocase of the inner mitochondrial membrane (TIM23 complex)
*TTR1*	0.9718	78.3	Decreased	mouse intravenous infection	[148]	Putative glutaredoxin; described as a glutathione reductase
*YML6*	0.9841	78.3	Decreased	mouse intravenous infection	[128]	Putative mitochondrial ribosomal protein
*C1_00510W_A*	0.9679	87	Absent	mouse intravenous infection	[128]	Ortholog(s) have 3-methyl-2-oxobutanoate hydroxymethyltransferase activity, role in pantothenate biosynthetic process, respiratory chain complex IV assembly
*C6_00920W_A*	0.5765	52.2	Decreased damage	reconstituted human epithelium	[149]	Putative mitochondrial intermembrane space protein

These types of bioinformatics predictions, and the genes identified, are a starting point to discover which specific processes conferred by mitochondria are important for virulence. Such genes can be good candidates for additional experiments. In species studied by a large research community, libraries of strains can be made for predicted mitochondrial proteins, by gene disruption to test phenotypes and fluorescent tagging of the proteins to confirm their localization. Such libraries can be used to screen for any change in the localization of tagged proteins under stressors, such as exposure to drugs, hypoxia, or mammalian body temperature. For the proteins that show sole mitochondrial localization under normal conditions, changes in localization to other organelles such as the nucleus, cell membrane or ER under stress can be examined. For the proteins that have dual localization under normal conditions, it can be observed if there is significant change in preferential localization to any one organelle or if there is diminished localization. Such experiments can define the mitochondrial components that are essential for virulence. This information will also help to provide a comprehensive picture of intracellular mitochondrial signaling networks required to cope with stress, and account for the strategies adopted by pathogens while interacting with the host.

In summary, at present there is evidence that the mitochondria are not merely a respiratory hub in fungal cells, but also a hub of activities that directly relate to the outcome of infections by pathogenic fungi. However, we are only beginning to define what aspects of the mitochondrial functions are specific to virulence. Ideally, gaining this knowledge will provide a framework for better therapies against medically prevalent fungi.

## References

[1] King N Amino acids and the mitochondria In: Schaffer SW, Suleiman MS, eds. Mitochondria: the Dynamic Organelle / Advances in Biochemistry in Health and Disease. New York, NY: Springer; 2007 151–66.

[2] Muhlenhoff U, Hoffmann B, Richter N, et al. Compartmentalization of iron between mitochondria and the cytosol and its regulation. Eur J Cell Biol. 2015;94:292–308. https://doi.org/10.1016/j.ejcb.2015.05.003. 26116073

[3] Ernster L, Schatz G Mitochondria: a historical review. J Cell Biol. 1981;91:227s–55s. https://doi.org/10.1083/jcb.91.3.227s. 7033239 PMC2112799

[4] Luft R, Ikkos D, Palmieri G, et al. A case of severe hypermetabolism of nonthyroid origin with a defect in the maintenance of mitochondrial respiratory control: a correlated clinical, biochemical, and morphological study. J Clin Invest. 1962;41:1776–804. https://doi.org/10.1172/JCI104637. 14467237 PMC291101

[5] Linnane AW, Saunders GW, Gingold EB, et al. The biogenesis of mitochondria. V. Cytoplasmic inheritance of erythromycin resistance in *Saccharomyces cerevisiae*. Proc Natl Acad Sci U S A. 1968;59:903–10. https://doi.org/10.1073/pnas.59.3.903. 5238670 PMC224776

[6] Holt IJ, Harding AE, Morgan-Hughes JA Deletions of muscle mitochondrial DNA in patients with mitochondrial myopathies. Nature. 1988;331:717–9. https://doi.org/10.1038/331717a0. 2830540

[7] Wallace DC, Singh G, Lott MT, et al. Mitochondrial DNA mutation associated with Leber's hereditary optic neuropathy. Science. 1988;242:1427–30. https://doi.org/10.1126/science.3201231. 3201231

[8] Green DR, Reed JC Mitochondria and apoptosis. Science. 1998;281:1309–12. https://doi.org/10.1126/science.281.5381.1309. 9721092

[9] Parker WD Jr., Boyson SJ, Parks JK Abnormalities of the electron transport chain in idiopathic Parkinson's disease. Ann Neurol. 1989;26:719–23. https://doi.org/10.1002/ana.410260606. 2557792

[10] Parker WD Jr. Cytochrome oxidase deficiency in Alzheimer's disease. Ann N Y Acad Sci. 1991;640:59–64. https://doi.org/10.1111/j.1749-6632.1991.tb00191.x. 1663716

[11] Sivitz WI, Yorek MA Mitochondrial dysfunction in diabetes: from molecular mechanisms to functional significance and therapeutic opportunities. Antioxid Redox Signal. 2010;12:537–77. https://doi.org/10.1089/ars.2009.2531. 19650713 PMC2824521

[12] Birsoy K, Wang T, Chen WW, et al. An essential role of the mitochondrial electron transport chain in cell proliferation is to enable aspartate synthesis. Cell. 2015;162:540–51. https://doi.org/10.1016/j.cell.2015.07.016. 26232224 PMC4522279

[13] Pellegrino MW, Nargund AM, Kirienko NV, et al. Mitochondrial UPR-regulated innate immunity provides resistance to pathogen infection. Nature. 2014;516:414–7. https://doi.org/10.1038/nature13818. 25274306 PMC4270954

[14] Hadwiger LA, Polashock J Fungal mitochondrial DNases: effectors with the potential to activate plant defenses in nonhost resistance. Phytopathology. 2013;103:81–90. https://doi.org/10.1094/PHYTO-04-12-0085-R. 23228145

[15] Luckhart S, Pakpour N, Giulivi C Host-pathogen interactions in malaria: cross-kingdom signaling and mitochondrial regulation. Curr Opin Immunol. 2015;36:73–9. https://doi.org/10.1016/j.coi.2015.07.002. 26210301 PMC4593738

[16] Rudel T, Kepp O, Kozjak-Pavlovic V Interactions between bacterial pathogens and mitochondrial cell death pathways. Nat Rev Microbiol. 2010;8:693–705. https://doi.org/10.1038/nrmicro2421. 20818415

[17] Makiuchi T, Nozaki T Highly divergent mitochondrion-related organelles in anaerobic parasitic protozoa. Biochimie. 2014;100:3–17. https://doi.org/10.1016/j.biochi.2013.11.018. 24316280

[18] Freibert S-A, Goldberg AV, Hacker C, et al. Evolutionary conservation and *in vitro* reconstitution of microsporidian iron-sulfur cluster biosynthesis. Nat Commun. 2017;8:13932. https://doi.org/10.1038/ncomms13932. 28051091 PMC5216125

[19] Ma H, Hagen F, Stekel DJ, et al. The fatal fungal outbreak on Vancouver Island is characterized by enhanced intracellular parasitism driven by mitochondrial regulation. Proc Natl Acad Sci U S A. 2009;106:12980–5. https://doi.org/10.1073/pnas.0902963106. 19651610 PMC2722359

[20] She X, Zhang L, Chen H, et al. Cell surface changes in the *Candida albicans* mitochondrial mutant *goa1*Δ are associated with reduced recognition by innate immune cells. Cell Microbiol. 2013;15:1572–84. https://doi.org/10.1111/cmi.12135. 23490206 PMC3738057

[21] Sun N, Fonzi W, Chen H, et al. Azole susceptibility and transcriptome profiling in *Candida albicans* mitochondrial electron transport chain complex I mutants. Antimicrob Agents Chemother. 2013;57:532–42. https://doi.org/10.1128/AAC.01520-12. 23147730 PMC3535965

[22] Bambach A, Fernandes MP, Ghosh A, et al. Goa1p of *Candida albicans* localizes to the mitochondria during stress and is required for mitochondrial function and virulence. Eukaryot Cell. 2009;8:1706–20. https://doi.org/10.1128/EC.00066-09. 19717740 PMC2772395

[23] Kretschmer M, Klose J, Kronstad JW Defects in mitochondrial and peroxisomal β-oxidation influence virulence in the maize pathogen *Ustilago maydis*. Eukaryot Cell. 2012;11:1055–66. https://doi.org/10.1128/EC.00129-12. 22707484 PMC3416065

[24] Olson Å, Stenlid J Plant pathogens: mitochondrial control of fungal hybrid virulence. Nature. 2001;411:438. 11373666 10.1038/35078147

[25] Mahlert M, Vogler C, Stelter K, et al. The *a2* mating-type-locus gene *lga2* of *Ustilago maydis* interferes with mitochondrial dynamics and fusion, partially in dependence on a Dnm1-like fission component. J Cell Sci. 2009;122:2402–12. https://doi.org/10.1242/jcs.039354. 19531588

[26] Du Y, Zhang H, Hong L, et al. Acetolactate synthases MoIlv2 and MoIlv6 are required for infection-related morphogenesis in *Magnaporthe oryzae*. Mol Plant Pathol. 2013;14:870–84. https://doi.org/10.1111/mpp.12053. 23782532 PMC6638861

[27] Foury F, Roganti T, Lecrenier N, et al. The complete sequence of the mitochondrial genome of *Saccharomyces cerevisiae*. FEBS Lett. 1998;440:325–31. https://doi.org/10.1016/S0014-5793(98)01467-7. 9872396

[28] Langkjaer RB, Casaregola S, Ussery DW, et al. Sequence analysis of three mitochondrial DNA molecules reveals interesting differences among *Saccharomyces* yeasts. Nucleic Acids Res. 2003;31:3081–91. https://doi.org/10.1093/nar/gkg423. 12799436 PMC162263

[29] Koszul R, Malpertuy A, Frangeul L, et al. The complete mitochondrial genome sequence of the pathogenic yeast *Candida* (*Torulopsis*) *glabrata*. FEBS Lett. 2003;534:39–48. https://doi.org/10.1016/S0014-5793(02)03749-3. 12527359

[30] Ma H, May RC Mitochondria and the regulation of hypervirulence in the fatal fungal outbreak on Vancouver Island. Virulence. 2010;1:197–201. https://doi.org/10.4161/viru.1.3.11053. 21178442 PMC3073246

[31] Joardar V, Abrams NF, Hostetler J, et al. Sequencing of mitochondrial genomes of nine *Aspergillus* and *Penicillium* species identifies mobile introns and accessory genes as main sources of genome size variability. BMC Genomics. 2012;13:698. https://doi.org/10.1186/1471-2164-13-698. 23234273 PMC3562157

[32] Brown GD, Denning DW, Gow NAR, et al. Hidden killers: human fungal infections. Sci Transl Med. 2012;4:165.rv13. https://doi.org/10.1126/scitranslmed.3004404. 23253612

[33] Calderone R, Li D, Traven A System-level impact of mitochondria on fungal virulence: to metabolism and beyond. FEMS Yeast Res. 2015;15:fov027. https://doi.org/10.1093/femsyr/fov027. 26002841 PMC4542695

[34] Li D, Calderone R Exploiting mitochondria as targets for the development of new antifungals. Virulence. 2017;8:159–68. https://doi.org/10.1080/21505594.2016.1188235. 27191707 PMC5354164

[35] Thangamani S, Maland M, Mohammad H, et al. Repurposing approach identifies auranofin with broad spectrum antifungal activity that targets Mia40-Erv1 pathway. Front Cell Infect Microbiol. 2017;7:4. https://doi.org/10.3389/fcimb.2017.00004. 28149831 PMC5241286

[36] Okamoto K, Shaw JM Mitochondrial morphology and dynamics in yeast and multicellular eukaryotes. Annu Rev Genet. 2005;39:503–36. https://doi.org/10.1146/annurev.genet.38.072902.093019. 16285870

[37] Youle RJ, van der Bliek AM Mitochondrial fission, fusion, and stress. Science. 2012;337:1062–5. https://doi.org/10.1126/science.1219855. 22936770 PMC4762028

[38] Neubauer M, Zhu Z, Penka M, et al. Mitochondrial dynamics in the pathogenic mold *Aspergillus fumigatus*: therapeutic and evolutionary implications. Mol Microbiol. 2015;98:930–45. https://doi.org/10.1111/mmi.13167. 26272083

[39] Hagen F, Khayhan K, Theelen B, et al. Recognition of seven species in the *Cryptococcus gattii/Cryptococcus neoformans* species complex. Fungal Genet Biol. 2015;78:16–48. https://doi.org/10.1016/j.fgb.2015.02.009. 25721988

[40] Byrnes EJ, 3rd Li W, Lewit Y, et al. Emergence and pathogenicity of highly virulent *Cryptococcus gattii* genotypes in the northwest United States. PLoS Pathog. 2010;6:e1000850. https://doi.org/10.1371/journal.ppat.1000850. 20421942 PMC2858702

[41] Voelz K, Johnston SA, Smith LM, et al. ‘Division of labour’ in response to host oxidative burst drives a fatal *Cryptococcus gattii* outbreak. Nat Commun. 2014;5:5194. https://doi.org/10.1038/ncomms6194. 25323068 PMC4208095

[42] Thomas E, Roman E, Claypool S, et al. Mitochondria influence CDR1 efflux pump activity, Hog1-mediated oxidative stress pathway, iron homeostasis, and ergosterol levels in *Candida albicans*. Antimicrob Agents Chemother. 2013;57:5580–99. https://doi.org/10.1128/AAC.00889-13. 23979757 PMC3811284

[43] Khan IA, Ning G, Liu X, et al. Mitochondrial fission protein MoFis1 mediates conidiation and is required for full virulence of the rice blast fungus *Magnaporthe oryzae*. Microbiol Res. 2015;178:51–8. https://doi.org/10.1016/j.micres.2015.06.002. 26302847

[44] Patkar RN, Ramos-Pamplona M, Gupta AP, et al. Mitochondrial β-oxidation regulates organellar integrity and is necessary for conidial germination and invasive growth in *Magnaporthe oryzae*. Mol Microbiol. 2012;86:1345–63. https://doi.org/10.1111/mmi.12060. 23043393

[45] Ulrich JT, Mathre DE Mode of action of oxathiin systemic fungicides. V. Effect on electron transport system of *Ustilago maydis* and *Saccharomyces cerevisiae*. J Bacteriol. 1972;110:628–32. 4336692 10.1128/jb.110.2.628-632.1972 PMC247458

[46] Walter H, Tobler H, Gribkov D, et al. Sedaxane, isopyrazam and solatenol: novel broad-spectrum fungicides inhibiting succinate dehydrogenase (SDH) – synthesis challenges and biological aspects. Chimia. 2015;69:425–34. https://doi.org/10.2533/chimia.2015.425. 28482975

[47] Bartlett DW, Clough JM, Godwin JR, et al. The strobilurin fungicides. Pest Manag Sci. 2002;58:649–62. https://doi.org/10.1002/ps.520. 12146165

[48] Sierotzki H, Scalliet G A review of current knowledge of resistance aspects for the next-generation succinate dehydrogenase inhibitor fungicides. Phytopathology. 2013;103:880–7. https://doi.org/10.1094/PHYTO-01-13-0009-RVW. 23593940

[49] Takahata S, Kubota N, Takei-Masuda N, et al. Mechanism of action of ME1111, a novel antifungal agent for topical treatment of onychomycosis. Antimicrob Agents Chemother. 2016;60:873–80. https://doi.org/10.1128/AAC.01790-15. 26596944 PMC4750688

[50] Oliver JD, Sibley GEM, Beckmann N, et al. F901318 represents a novel class of antifungal drug that inhibits dihydroorotate dehydrogenase. Proc Natl Acad Sci USA. 2016;113:12809–14. https://doi.org/10.1073/pnas.1608304113. PMC5111691 27791100

[51] Vincent BM, Langlois J-B, Srinivas R, et al. A fungal-selective cytochrome *bc*_1_ inhibitor impairs virulence and prevents the evolution of drug resistance. Cell Chem Biol. 2016;23:978–91. https://doi.org/10.1016/j.chembiol.2016.06.016. 27524297 PMC5159300

[52] Guan G, Wang H, Liang W, et al. The mitochondrial protein Mcu1 plays important roles in carbon source utilization, filamentation, and virulence in *Candida albicans*. Fungal Genet Biol. 2015;81:150–9. https://doi.org/10.1016/j.fgb.2015.01.006. 25626172

[53] Xu J, Yan Z, Guo H Divergence, hybridization, and recombination in the mitochondrial genome of the human pathogenic yeast *Cryptococcus gattii*. Mol Ecol. 2009;18:2628–42.https://doi.org/10.1111/j.1365-294X.2009.04227.x. 19457185

[54] Bovers M, Hagen F, Kuramae EE, et al. Promiscuous mitochondria in *Cryptococcus gattii*. FEMS Yeast Res. 2009;9:489–503.https://doi.org/10.1111/j.1567-1364.2009.00494.x. 19281475

[55] Farrer RA, Desjardins CA, Sakthikumar S, et al. Genome evolution and innovation across the four major lineages of *Cryptococcus gattii*. mBio. 2015;6:e00868–15. https://doi.org/10.1128/mBio.00868-15. 26330512 PMC4556806

[56] Voelz K, Ma H, Phadke S, et al. Transmission of Hypervirulence traits via sexual reproduction within and between lineages of the human fungal pathogen *Cryptococcus gattii*. PLoS Genet. 2013;9:e1003771. https://doi.org/10.1371/journal.pgen.1003771. 24039607 PMC3764205

[57] Fraser JA, Giles SS, Wenink EC, et al. Same-sex mating and the origin of the Vancouver Island *Cryptococcus gattii* outbreak. Nature. 2005;437:1360–4. https://doi.org/10.1038/nature04220. 16222245

[58] Fu C, Sun S, Billmyre RB, et al. Unisexual versus bisexual mating in *Cryptococcus neoformans*: Consequences and biological impacts. Fungal Genet Biol. 2015;78:65–75. https://doi.org/10.1016/j.fgb.2014.08.008. 25173822 PMC4344436

[59] Phadke SS, Feretzaki M, Clancey SA, et al. Unisexual reproduction of *Cryptococcus gattii*. PLoS One. 2014;9:e111089. https://doi.org/10.1371/journal.pone.0111089. 25337713 PMC4206507

[60] O'Gorman CM, Fuller HT, Dyer PS Discovery of a sexual cycle in the opportunistic fungal pathogen *Aspergillus fumigatus*. Nature. 2009;457:471–4. https://doi.org/10.1038/nature07528. 19043401

[61] Losada L, Sugui JA, Eckhaus MA, et al. Genetic analysis using an isogenic mating pair of *Aspergillus fumigatus* identifies Aazole resistance genes and lack of *MAT* locus's role in virulence. PLoS Pathog. 2015;11:e1004834. https://doi.org/10.1371/journal.ppat.1004834. 25909486 PMC4409388

[62] Forche A, Alby K, Schaefer D, et al. The parasexual cycle in *Candida albicans* provides an alternative pathway to meiosis for the formation of recombinant strains. PLoS Biol. 2008;6:e110. https://doi.org/10.1371/journal.pbio.0060110. 18462019 PMC2365976

[63] Bennett RJ, Johnson AD Completion of a parasexual cycle in *Candida albicans* by induced chromosome loss in tetraploid strains. EMBO J. 2003;22:2505–15. https://doi.org/10.1093/emboj/cdg235. 12743044 PMC155993

[64] Jacobsen MD, Rattray AMJ, Gow NAR, et al. Mitochondrial haplotypes and recombination in *Candida albicans*. Med Mycol. 2008;46:647–54. https://doi.org/10.1080/13693780801986631. 18608923

[65] Valach M, Pryszcz LP, Tomaska L, et al. Mitochondrial genome variability within the *Candida parapsilosis* species complex. Mitochondrion. 2012;12:514–9. https://doi.org/10.1016/j.mito.2012.07.109. 22824459

[66] Anderson JB, Wickens C, Khan M, et al. Infrequent genetic exchange and recombination in the mitochondrial genome of *Candida albicans*. J Bacteriol. 2001;183:865–72. https://doi.org/10.1128/JB.183.3.865-872.2001. 11208783 PMC94952

[67] Pryszcz LP, Németh T, Saus E, et al. The genomic aftermath of hybridization in the opportunistic pathogen *Candida metapsilosis*. PLoS Genet. 2015;11:e1005626. https://doi.org/10.1371/journal.pgen.1005626. 26517373 PMC4627764

[68] Hamari Z, Toth B, Beer Z, et al. Interpretation of intraspecific variability in mtDNAs of *Aspergillus niger* strains and rearrangement of their mtDNAs following mitochondrial transmissions. FEMS Microbiol Lett. 2003;221:63–71. https://doi.org/10.1016/S0378-1097(03)00165-4. 12694912

[69] Yan Z, Xu J Mitochondria are inherited from the *MAT*a parent in crosses of the basidiomycete fungus *Cryptococcus neoformans*. Genetics. 2003;163:1315–25. 12702677 10.1093/genetics/163.4.1315 PMC1462512

[70] Gyawali R, Lin X Prezygotic and postzygotic control of uniparental mitochondrial DNA inheritance in *Cryptococcus neoformans*. mBio. 2013;4:e00112–13. https://doi.org/10.1128/mBio.00112-13. 23611907 PMC3638309

[71] Zhu P, Zhai B, Lin X, et al. Congenic strains for genetic analysis of virulence traits in *Cryptococcus gattii*. Infect Immun. 2013;81:2616–25. https://doi.org/10.1128/IAI.00018-13. 23670558 PMC3697594

[72] Shakya VPS, Idnurm A Sex determination directs uniparental mitochondrial inheritance in *Phycomyces*. Eukaryot Cell. 2014;13:186–9. https://doi.org/10.1128/EC.00203-13. 24243797 PMC3910969

[73] Parikh VS, Morgan MM, Scott R, et al. The mitochondrial genotype can influence nuclear gene expression in yeast. Science. 1987;235:576–80. https://doi.org/10.1126/science.3027892. 3027892

[74] Liao X, Butow RA *RTG1* and *RTG2*: two yeast genes required for a novel path of communication from mitochondria to the nucleus. Cell. 1993;72:61–71. https://doi.org/10.1016/0092-8674(93)90050-Z. 8422683

[75] Hallstrom TC, Moye-Rowley WS Multiple signals from dysfunctional mitochondria activate the pleiotropic drug resistance pathway in *Saccharomyces cerevisiae*. J Biol Chem. 2000;275:37347–56. https://doi.org/10.1074/jbc.M007338200. 10980204

[76] Moye-Rowley WS Retrograde regulation of multidrug resistance in *Saccharomyces cerevisiae*. Gene. 2005;354:15–21. https://doi.org/10.1016/j.gene.2005.03.019. 15896930

[77] Traven A, Wong JMS, Xu D, et al. Interorganellar communication. Altered nuclear gene expression profiles in a yeast mitochondrial DNA mutant. J Biol Chem. 2001;276:4020–7. https://doi.org/10.1074/jbc.M006807200. 11054416

[78] Spinazzola A, Zeviani M Mitochondrial diseases: a cross-talk between mitochondrial and nuclear genomes. Adv Exp Med Biol. 2009;652:69–84. https://doi.org/10.1007/978-90-481-2813-6_6. 20225020

[79] Zhang X, Moye-Rowley WS *Saccharomyces cerevisiae* multidrug resistance gene expression inversely correlates with the status of the F_0_ component of the mitochondrial ATPase. J Biol Chem. 2001;276:47844–52. https://doi.org/10.1074/jbc.M106285200. 11602584

[80] Shingu-Vazquez M, Traven A Mitochondria and fungal pathogenesis: drug tolerance, virulence, and potential for antifungal therapy. Eukaryot Cell. 2011;10:1376–83. https://doi.org/10.1128/EC.05184-11. 21926328 PMC3209048

[81] Brun S, Bergès T, Poupard P, et al. Mechanisms of azole resistance in petite mutants of *Candida glabrata*. Antimicrob Agents Chemother. 2004;48:1788–96. https://doi.org/10.1128/AAC.48.5.1788-1796.2004. 15105136 PMC400549

[82] Paul S, Schmidt JA, Moye-Rowley WS Regulation of the CgPdr1 transcription factor from the pathogen *Candida glabrata*. Eukaryot Cell. 2011;10:187–97. https://doi.org/10.1128/EC.00277-10. 21131438 PMC3067410

[83] Tsai H-F, Krol AA, Sarti KE, et al. *Candida glabrata PDR1*, a transcriptional regulator of a pleiotropic drug resistance network, mediates azole resistance in clinical isolates and petite mutants. Antimicrob Agents Chemother. 2006;50:1384–92. https://doi.org/10.1128/AAC.50.4.1384-1392.2006. 16569856 PMC1426987

[84] Paul S, Bair TB, Moye-Rowley WS Identification of genomic binding sites for *Candida glabrata* Pdr1 transcription factor in wild-type and ρ^0^ cells. Antimicrob Agents Chemother. 2014;58:6904–12. https://doi.org/10.1128/AAC.03921-14. 25199772 PMC4249425

[85] Ferrari S, Ischer F, Calabrese D, et al. Gain of function mutations in *CgPDR1* of *Candida glabrata* not only mediate antifungal resistance but also enhance virulence. PLoS Pathog. 2009;5:e1000268. https://doi.org/10.1371/journal.ppat.1000268. 19148266 PMC2607542

[86] Ferrari S, Sanguinetti M, De Bernardis F, Torelli R, et al. Loss of mitochondrial functions associated with azole resistance in *Candida glabrata* results in enhanced virulence in mice. Antimicrob Agents Chemother. 2011;55:1852–60. https://doi.org/10.1128/AAC.01271-10. 21321146 PMC3088236

[87] Devaux F, Carvajal E, Moye-Rowley S, et al. Genome-wide studies on the nuclear PDR3-controlled response to mitochondrial dysfunction in yeast. FEBS Lett. 2002;515:25–8. https://doi.org/10.1016/S0014-5793(02)02387-6. 11943188

[88] Panwar SL, Moye-Rowley WS Long chain base tolerance in *Saccharomyces cerevisiae* is induced by retrograde signals from the mitochondria. J Biol Chem. 2006;281:6376–84. https://doi.org/10.1074/jbc.M512115200. 16407254

[89] Gulshan K, Schmidt JA, Shahi P, et al. Evidence for the bifunctional nature of mitochondrial phosphatidylserine decarboxylase: role in Pdr3-dependent retrograde regulation of *PDR5* expression. Mol Cell Biol. 2008;28:5851–64. https://doi.org/10.1128/MCB.00405-08. 18644857 PMC2547020

[90] Batova M, Borecka-Melkusova S, Simockova M, et al. Functional characterization of the *CgPGS1* gene reveals a link between mitochondrial phospholipid homeostasis and drug resistance in *Candida glabrata*. Curr Genet. 2008;53:313–22. https://doi.org/10.1007/s00294-008-0187-9. 18343926

[91] Singh A, Yadav V, Prasad R Comparative lipidomics in clinical isolates of *Candida albicans* reveal crosstalk between mitochondria, cell wall integrity and azole resistance. PLoS One. 2012;7:e39812. https://doi.org/10.1371/journal.pone.0039812. 22761908 PMC3384591

[92] López-Marqués RL, Poulsen LR, Bailly A, et al. Structure and mechanism of ATP-dependent phospholipid transporters. Biochim Biophys Acta. 2015;1850:461–75. https://doi.org/10.1016/j.bbagen.2014.04.008. 24746984

[93] Bromley M, Johns A, Davies E, et al. Mitochondrial complex I Is a global regulator of secondary metabolism, virulence and azole sensitivity in fungi. PLoS One. 2016;11:e0158724. https://doi.org/10.1371/journal.pone.0158724. 27438017 PMC4954691

[94] Vandeputte P, Tronchin G, Rocher F, et al. Hypersusceptibility to azole antifungals in a clinical isolate of *Candida glabrata* with reduced aerobic growth. Antimicrob Agents Chemother. 2009;53:3034–41. https://doi.org/10.1128/AAC.01384-08. 19380598 PMC2704708

[95] Epstein CB, Waddle JA, Hale IV W, et al. Genome-wide responses to mitochondrial dysfunction. Mol Biol Cell. 2001;12:297–308. https://doi.org/10.1091/mbc.12.2.297. 11179416 PMC30944

[96] Girvan HM, Munro AW Heme sensor proteins. J Biol Chem. 2013;288:13194–203. https://doi.org/10.1074/jbc.R112.422642. 23539616 PMC3650359

[97] Cresnar B, Petric S Cytochrome P450 enzymes in the fungal kingdom. Biochim Biophys Acta. 2011;1814:29–35. https://doi.org/10.1016/j.bbapap.2010.06.020. 20619366

[98] Balding PR, Porro CS, McLean KJ, et al. How do azoles inhibit cytochrome P450 enzymes? A density functional study. J Phys Chem A. 2008;112:12911–8. https://doi.org/10.1021/jp802087w. 18563875

[99] Lupetti A, Danesi R, Campa M, et al. Molecular basis of resistance to azole antifungals. Trends Mol Med. 2002;8:76–81. https://doi.org/10.1016/S1471-4914(02)02280-3. 11815273

[100] Hosogaya N, Miyazaki T, Nagi M, et al. The heme-binding protein Dap1 links iron homeostasis to azole resistance via the P450 protein Erg11 in *Candida glabrata*. FEMS Yeast Res. 2013;13:411–21. https://doi.org/10.1111/1567-1364.12043. 23496820

[101] Song J, Zhai P, Zhang Y, et al. The *Aspergillus fumigatus* damage resistance protein family coordinately regulates ergosterol biosynthesis and azole susceptibility. mBio. 2016;7:e01919–15. https://doi.org/10.1128/mBio.01919-15. 26908577 PMC4791848

[102] Mallory JC, Crudden G, Johnson BL, et al. Dap1p, a heme-binding protein that regulates the cytochrome P450 protein Erg11p/Cyp51p in *Saccharomyces cerevisiae*. Mol Cell Biol. 2005;25:1669–79. https://doi.org/10.1128/MCB.25.5.1669-1679.2005. 15713626 PMC549369

[103] Hughes AL, Powell DW, Bard M, et al. Dap1/PGRMC1 binds and regulates cytochrome P450 enzymes. Cell Metab. 2007;5:143–9. https://doi.org/10.1016/j.cmet.2006.12.009. 17276356

[104] Long N, Xu X, Qian H, et al. A putative mitochondrial iron transporter MrsA in *Aspergillus fumigatus* plays important roles in azole-, oxidative stress responses and virulence. Front Microbiol. 2016;7:716. https://doi.org/10.3389/fmicb.2016.00716. 27433157 PMC4922219

[105] Schrettl M, Kim HS, Eisendle M, et al. SreA-mediated iron regulation in *Aspergillus fumigatus*. Mol Microbiol. 2008;70:27–43. https://doi.org/10.1111/j.1365-2958.2008.06376.x. 18721228 PMC2610380

[106] Stehling O, Lill R The role of mitochondria in cellular iron-sulfur protein biogenesis: mechanisms, connected processes, and diseases. Cold Spring Harb Perspect Biol. 2013;5:a011312. https://doi.org/10.1101/cshperspect.a011312. 23906713 PMC3721283

[107] Braymer JJ, Lill R Iron-sulfur cluster biogenesis and trafficking in mitochondria. J Biol Chem. 2017;292:12754–63. https://doi.org/10.1074/jbc.R117.787101. 28615445 PMC5546016

[108] Buschlen S, Amillet J-M, Guiard B, et al. The *S. cerevisiae* HAP complex, a key regulator of mitochondrial function, coordinates nuclear and mitochondrial gene expression. Comp Funct Genomics. 2003;4:37–46. https://doi.org/10.1002/cfg.254. 18629096 PMC2447382

[109] McNabb DS, Xing Y, Guarente L Cloning of yeast *HAP5*: a novel subunit of a heterotrimeric complex required for CCAAT binding. Genes Dev. 1995;9:47–58. https://doi.org/10.1101/gad.9.1.47. 7828851

[110] Tanaka A, Kato M, Nagase T, et al. Isolation of genes encoding novel transcription factors which interact with the Hap complex from *Aspergillus* species. Biochim Biophys Acta. 2002;1576:176–82. https://doi.org/10.1016/S0167-4781(02)00286-5. 12031499

[111] Schrettl M, Beckmann N, Varga J, et al. HapX-mediated adaption to iron starvation is crucial for virulence of *Aspergillus fumigatus*. PLoS Pathog. 2010;6:e1001124. https://doi.org/10.1371/journal.ppat.1001124. 20941352 PMC2947994

[112] Hortschansky P, Eisendle M, Al-Abdallah Q, et al. Interaction of HapX with the CCAAT-binding complex—a novel mechanism of gene regulation by iron. EMBO J. 2007;26:3157–68. https://doi.org/10.1038/sj.emboj.7601752. 17568774 PMC1914100

[113] Hsu P-C, Yang C-Y, Lan C-Y *Candida albicans* Hap43 is a repressor induced under low-iron conditions and is essential for iron-responsive transcriptional regulation and virulence. Eukaryot Cell. 2011;10:207–25. https://doi.org/10.1128/EC.00158-10. 21131439 PMC3067405

[114] Jung WH, Saikia S, Hu G, et al. HapX positively and negatively regulates the transcriptional response to iron deprivation in *Cryptococcus neoformans*. PLoS Pathog. 2010;6:e1001209. https://doi.org/10.1371/journal.ppat.1001209. 21124817 PMC2991262

[115] López-Berges MS, Capilla J, Turrà D, et al. HapX-mediated iron homeostasis is essential for rhizosphere competence and virulence of the soilborne pathogen *Fusarium oxysporum*. Plant Cell. 2012;24:3805–22. https://doi.org/10.1105/tpc.112.098624. 22968717 PMC3480304

[116] Caza M, Hu G, Price M, et al. The zinc finger protein Mig1 regulates mitochondrial function and azole drug susceptibility in the pathogenic fungus *Cryptococcus neoformans*. mSphere. 2016;https://doi.org/10.1128/mSphere.00080-15. PMC4863601 27303693

[117] Garcia-Santamarina S, Uzarska MA, Festa RA, et al. *Cryptococcus neoformans* iron-sulfur protein biogenesis machinery is a novel layer of protection against Cu stress. mBio. 2017;8:e01742–17. https://doi.org/10.1128/mBio.01742-17. 29089435 PMC5666163

[118] Qu Y, Jelicic B, Pettolino F, et al. Mitochondrial sorting and assembly machinery subunit Sam37 in *Candida albicans*: insight into the roles of mitochondria in fitness, cell wall integrity, and virulence. Eukaryot Cell. 2012;11:532–44. https://doi.org/10.1128/EC.05292-11. 22286093 PMC3318305

[119] Neupert W, Herrmann JM Translocation of proteins into mitochondria. Annu Rev Biochem. 2007;76:723–49. https://doi.org/10.1146/annurev.biochem.76.052705.163409. 17263664

[120] Chacinska A, Koehler CM, Milenkovic D, et al. Importing mitochondrial proteins: machineries and mechanisms. Cell. 2009;138:628–44. https://doi.org/10.1016/j.cell.2009.08.005. 19703392 PMC4099469

[121] Ellenrieder L, Opaliński Ł, Becker L, et al. Separating mitochondrial protein assembly and endoplasmic reticulum tethering by selective coupling of Mdm10. Nat Commun. 2016;7:13021. https://doi.org/10.1038/ncomms13021. 27721450 PMC5476798

[122] Dagley MJ, Gentle IE, Beilharz TH, et al. Cell wall integrity is linked to mitochondria and phospholipid homeostasis in *Candida albicans* through the activity of the post-transcriptional regulator Ccr4-Pop2. Mol Microbiol. 2011;79:968–89. https://doi.org/10.1111/j.1365-2958.2010.07503.x. 21299651

[123] Hewitt VL, Heinz E, Shingu-Vazquez M, et al. A model system for mitochondrial biogenesis reveals evolutionary rewiring of protein import and membrane assembly pathways. Proc Natl Acad Sci U S A. 2012;109:E3358–66. https://doi.org/10.1073/pnas.1206345109. 23151513 PMC3523872

[124] Malhotra JD, Kaufman RJ ER stress and its functional link to mitochondria: role in cell survival and death. Cold Spring Harb Perspect Biol. 2011;3:a004424. https://doi.org/10.1101/cshperspect.a004424. 21813400 PMC3181038

[125] Rainbolt TK, Saunders JM, Wiseman RL Stress-responsive regulation of mitochondria through the ER unfolded protein response. Trends Endocrinol Metab. 2014;25:528–37. https://doi.org/10.1016/j.tem.2014.06.007. 25048297

[126] Kornmann B, Currie E, Collins SR, et al. An ER-mitochondria tethering complex revealed by a synthetic biology screen. Science. 2009;325:477–81. https://doi.org/10.1126/science.1175088. 19556461 PMC2933203

[127] Tucey TM, Verma-Gaur J, Nguyen J, et al. The endoplasmic reticulum-mitochondrion tether ERMES orchestrates fungal immune evasion, illuminating inflammasome responses to hyphal signals. mSphere. 2016;1:e00074–16. https://doi.org/10.1128/mSphere.00074-16. PMC4888881 27303738

[128] Becker JM, Kauffman SJ, Hauser M, et al. Pathway analysis of *Candida albicans* survival and virulence determinants in a murine infection model. Proc Natl Acad Sci U S A. 2010;107:22044–9. https://doi.org/10.1073/pnas.1009845107. 21135205 PMC3009777

[129] Friedman JR, Lackner LL, West M, et al. ER tubules mark sites of mitochondrial division. Science. 2011;334:358–62. https://doi.org/10.1126/science.1207385. 21885730 PMC3366560

[130] Michel AH, Kornmann B The ERMES complex and ER-mitochondria connections. Biochem Soc Trans. 2012;40:445–50. https://doi.org/10.1042/BST20110758. 22435828

[131] Geißel B, Penka M, Neubauer M, et al. The ER-mitochondria encounter structure contributes to hyphal growth, mitochondrial morphology and virulence of the pathogenic mold *Aspergillus fumigatus*. Int J Med Microbiol. 2017;307:37–43. https://doi.org/10.1016/j.ijmm.2016.11.005. 27939177

[132] Jo A, Ham S, Lee GH, et al. Efficient mitochondrial genome editing by CRISPR/Cas9. Biomed Res Int. 2015;2015:305716. https://doi.org/10.1155/2015/305716. 26448933 PMC4581504

[133] Verma S, Idnurm A The Uve1 endonuclease is regulated by the white collar complex to protect *Cryptococcus neoformans* from UV damage. PLoS Genet. 2013;9:e1003769. https://doi.org/10.1371/journal.pgen.1003769. 24039606 PMC3764193

[134] Rhodes J, Desjardins CA, Sykes SM, et al. Tracing genetic exchange and biogeography of *Cryptococcus neoformans* var. *grubii* at the global population level. Genetics. 2017;207:327–46. https://doi.org/10.1534/genetics.117.203836. 28679543 PMC5586382

[135] Ianiri G, Idnurm A Essential gene discovery in the basidiomycete *Cryptococcus neoformans* for antifungal drug target prioritization. mBio. 2015;6:e02334–14. https://doi.org/10.1128/mBio.02334-14. 25827419 PMC4453551

[136] Scheckhuber CQ, Erjavec N, Tinazli A, et al. Reducing mitochondrial fission results in increased life span and fitness of two fungal ageing models. Nat Cell Biol. 2007;9:99–105. https://doi.org/10.1038/ncb1524. 17173038

[137] Scheckhuber CQ, Osiewacz HD *Podospora anserina*: a model organism to study mechanisms of healthy ageing. Mol Genet Genomics. 2008;280:365–74. https://doi.org/10.1007/s00438-008-0378-6. 18797929

[138] Bouklas T, Pechuan X, Goldman DL, et al. Old *Cryptococcus neoformans* cells contribute to virulence in chronic cryptococcosis. mBio. 2013;4:e00455–13. https://doi.org/10.1128/mBio.00455-13. 23943761 PMC3747583

[139] Bouklas T, Alonso-Crisóstomo L, Székely T Jr., et al. Generational distribution of a *Candida glabrata* population: resilient old cells prevail, while younger cells dominate in the vulnerable host. PLoS Pathog. 2017;13:e1006355. https://doi.org/10.1371/journal.ppat.1006355. 28489916 PMC5440053

[140] Nakai K, Kanehisa M A knowledge base for predicting protein localization sites in eukaryotic cells. Genomics. 1992;14:897–911. https://doi.org/10.1016/S0888-7543(05)80111-9. 1478671 PMC7134799

[141] Claros MG, Vincens P Computational method to predict mitochondrially imported proteins and their targeting sequences. Eur J Biochem. 1996;241:779–86. https://doi.org/10.1111/j.1432-1033.1996.00779.x. 8944766

[142] Bassilana M, Blyth J, Arkowitz RA Cdc24, the GDP-GTP exchange factor for Cdc42, is required for invasive hyphal growth of *Candida albicans*. Eukaryot Cell. 2003;2:9–18. https://doi.org/10.1128/EC.2.1.9-18.2003. 12582118 PMC141177

[143] Cleary IA, Lazzell AL, Monteagudo C, et al. *BRG1* and *NRG1* form a novel feedback circuit regulating *Candida albicans* hypha formation and virulence. Mol Microbiol. 2012;85:557–73. https://doi.org/10.1111/j.1365-2958.2012.08127.x. 22757963 PMC3402693

[144] Kingsbury JM, McCusker JH Cytocidal amino acid starvation of *Saccharomyces cerevisiae* and *Candida albicans* acetolactate synthase (*ilv2*Δ) mutants is influenced by the carbon source and rapamycin. Microbiology. 2010;156:929–39. https://doi.org/10.1099/mic.0.034348-0. 20019084 PMC2841795

[145] Gabriel I, Kur K, Laforce-Nesbitt SS, et al. Phenotypic consequences of *LYS4* gene disruption in *Candida albicans*. Yeast. 2014;31:299–308. https://doi.org/10.1002/yea.3021. 24898203

[146] Li Y, Su C, Mao X, et al. Roles of *Candida albicans* Sfl1 in hyphal development. Eukaryot Cell. 2007;6:2112–21. https://doi.org/10.1128/EC.00199-07. 17715361 PMC2168412

[147] Jackson BE, Wilhelmus KR, Mitchell BM Genetically regulated filamentation contributes to *Candida albicans* virulence during corneal infection. Microb Pathog. 2007;42:88–93. https://doi.org/10.1016/j.micpath.2006.11.005. 17241762 PMC1892154

[148] Chaves GM, Bates S, MacCallum DM, et al. *Candida albicans GRX2*, encoding a putative glutaredoxin, is required for virulence in a murine model. Genet Mol Res. 2007;6:1051–63. 18273798

[149] Zakikhany K, Naglik JR, Schmidt-Westhausen A, et al. *In vivo* transcript profiling of *Candida albicans* identifies a gene essential for interepithelial dissemination. Cell Microbiol. 2007;9:2938–54. https://doi.org/10.1111/j.1462-5822.2007.01009.x. 17645752

[150] Skrzypek MS, Binkley J, Binkley G, et al. The *Candida* Genome Database (CGD): incorporation of assembly 22, systematic identifiers and visualization of high throughput sequencing data. Nucleic Acids Res. 2017;45:D592–D6. https://doi.org/10.1093/nar/gkw924. 27738138 PMC5210628

